# Drivers of coffee liking: Effect of physicochemical characteristics and aromatic profile on consumers’ acceptability of mono‐origin and mono‐variety coffees

**DOI:** 10.1111/1750-3841.16323

**Published:** 2022-09-16

**Authors:** Nicola Condelli, Nazarena Cela, Maria Di Cairano, Teresa Scarpa, Luigi Milella, Roberta Ascrizzi, Guido Flamini, Fernanda Galgano

**Affiliations:** ^1^ School of Agricultural, Forestry, Food and Environmental Sciences (SAFE) University of Basilicata Potenza Italy; ^2^ Department of Science University of Basilicata Potenza Italy; ^3^ Department of Pharmacy University of Pisa Pisa Italy

**Keywords:** CATA (check all that apply), volatiles, acceptability, gas chromatography, arabica, robusta

## Abstract

Few studies have investigated at the same time how physicochemical, volatile, and sensory characteristics affect coffee liking. The aim of this study is to evaluate the influence of geographical origin and variety on physicochemical parameters and volatile compounds composition of mono‐origin and mono‐variety coffees. Check‐all‐that‐apply (CATA) method was used with the aim of identifying the drivers of coffee liking. Moisture content, bulk density, solubility index, color parameters, and phenols and caffeine content were useful parameters for discriminating Robusta from Arabica variety, but not samples from different origins. The hierarchical cluster and principal component analyses performed on the headspace compositions showed a quite sharp ability to group the samples based on their variety. Based on CATA results, roasted attribute, related to the presence of pyrazines, was considered a positive driver of coffee liking unlike grassy and acidic attributes (associated to the presence of acids and aldehydes, respectively). Findings from this study can be a useful tool for coffee manufacturers for a winning market strategy, helping them in the choice of the most suitable raw materials and process conditions in order to produce a well‐balanced beverage by enhancing all the possible positive drivers of acceptability and reducing the negative ones.

## 1 INTRODUCTION

Coffee is one of the most important international trade products, second only to crude oil. On average, 2.25 billion cups of coffee per day are consumed around the world (Kingsley et al., 2018). The investigation of factors influencing consumer acceptability of coffee is therefore an interesting aspect for coffee manufacturers. Each step of the coffee‐production process influences the quality of the final product, such as coffee varieties, geographical origin, pedoclimatic conditions, coffee cherry maturity, method of harvesting, drying, selection, and calibration of coffee beans, storage conditions, and roasting conditions (Ascrizzi & Flamini, 2020). In addition, the brewing method plays a crucial role in characteristics of the final product, for example, the blend of roasted coffee, grinding grade, dose of ground powder, temperature and pressure of water used for the extraction, and serving conditions (Giacalone et al., 2019; Seninde & Chambers, 2020).

Sensory properties of coffee, mainly flavor attributes, have a significant influence on consumers’ liking (Seninde & Chambers, 2020). Coffee is one of the most aromatic food products, with more than 900 aroma compounds identified (Akiyama et al., 2007; Seninde & Chambers, 2020; Toledo et al., 2016). They belong to a wide variety of chemical functional groups: the most relevant are aldehydes, ketones, phenols, sulfur compounds, pyrazines, pyridines, pyrroles, hydrocarbons, acids, anhydrides, esters, alcohols, and furans. Some of these compounds have a relevant influence on coffee flavor, such as acetic acid, 2‐methylpyrazine, furfural, 2‐furfuryl alcohol, 2,6‐dimethylpyrazine, and 5‐methylfurfural, and they are also used for distinguishing coffees based on variety and geographical origin (Toledo et al., 2016). Analysis of headspace of Robusta and Arabica samples by gas‐chromatography/olfactometry showed that there are specific aromatic compounds more present in some cultivars compared to others. For example, 2‐methylisoborneol is a volatile compound characteristic of the Robusta variety, by conferring earthy notes to the final product. In fact, each volatile compound provides a particular descriptive note: in Robusta coffees, the most perceived ones are spicy, earthy, roasty, whereas caramel and sweet are characteristic of Arabica ones. This difference increases in the brew, because the extraction process enhances the number of water‐soluble compounds, responsible for the typical caramel‐like and sweet notes perceived in Arabica coffees. The same occurs for pyrazines and guaiacols, linked to spicy and earthy notes in the Robusta samples (Blank et al., 1991).

Nonvolatile compounds also affect coffee flavor, as well as taste and mouthfeel of the final beverage: caffeine imparts bitterness and body; chlorogenic acids provide astringency and, as a consequence of the formation of lactones following reaction with quinic acid during roasting process, enhance bitterness; amino acids content influences the development of melanoidins through Maillard reaction, brownish compounds contributing to coffee color (Seninde & Chambers, 2020), as well as of pyrazines, key aroma‐active compounds in coffee (Ascrizzi & Flamini, 2020).

A different chemical composition of the coffee beans results in differences in the perceived sensory quality. Since coffee is one of the most consumed beverages worldwide, understanding the factors influencing its quality and how they are related to consumer acceptability is a crucial key, in order to provide the manufacturers with information on how to select the optimal raw material and to standardize and control the entire production process and comply with customers’ requests. For example, it is well known that Robusta varieties have a higher caffeine content than Arabica ones (Gaibor et al., 2020). Understanding if bitterness, mainly related to caffeine content, has a positive correlation with coffee liking is useful for coffee manufactures to develop the ideal blend of coffee.

Quantitative descriptive analysis (QDA) method is a sensory descriptive test commonly used to measure sensory characteristics of a product, useful for a quality control or to give more information to the consumer. Despite this, QDA methods require a trained panel; therefore, they are considered time‐consuming and quite expensive by food companies with limited consumer research budgets. For this purpose, rapid sensory tests have been developed in recent years to provide faster information about sensory perception of food products directly by customers and to identify characteristics driving consumers’ acceptability. One of them is the check‐all‐that‐apply (CATA) questionnaire (Ares et al., 2014), considered as a good alternative to QDA in determining specific sensory attributes of a product even if rapid sensory tests cannot replace QDA if the goal is to obtain a sensory profile with high accuracy (Hunaefi et al., 2020).

Several studies report a comprehensive characterization from both chemical‐analytical and sensorial perspectives on food matrices such as beer (Bauwens et al., 2021; da Costa Jardim et al., 2018; Mascia et al., 2014), wine (Han et al., 2022; Pérez‐Navarro et al., 2019; Sánchez‐Palomo et al., 2019), and olive oil (El Riachy et al., 2018; Procida et al., 2016; Zago et al., 2019). However, few studies correlate physicochemical, volatile, and sensory data (in terms of consumers’ acceptability) of the coffee together with an assessment of effect of variety and geographical origin. This study aimed to establish how geographical origin and variety affect physicochemical parameters and volatile compounds composition of mono‐origin and mono‐variety coffees and to describe a possible correlation of these factors with the overall liking. The CATA method was used with the aim of identifying the main drivers influencing overall liking of mono‐variety and mono‐origin coffee samples.

## 2 MATERIALS AND METHODS

### 2.1 Coffee samples formulation and preparation

Coffee beans were supplied by Escaffè srl (Oppido Lucano, Potenza, Italy). Twenty coffee samples (Table S1) were characterized in this study, according to their physicochemical characteristics. In total, 9 samples belonged to *Coffea arabica* L. (Arabica) (A1–A9), 7 to *Coffea canephora* Pierre ex A.Froehner (Robusta) (R1–R7), and the remainder were commercial blends (GC, GD, PH, PV). The latter were selected among the most consumed commercial brands by panelists. Six coffee samples came from Africa (A1, A2, R1, R4, R5, R7), four samples from America (A3, A4, A5, A6), and six samples were grown in Asian countries (A7, A8, A9, R2, R3, R6). The information on geographical origin was not available on blend samples, so they were grouped into “commercial” origin. Coffee beans were roasted at 245°C for 15 min in a pilot plant roaster (Escaffè srl, Oppido Lucano, Potenza, Italy). The same roasting degree was kept for all samples in order to avoid the variations in physicochemical and sensory characteristics due to this process. Coffee samples were grounded with a “Rancilio Rocky” coffee grinder (Rancilio Group spa, Milan, Italy) consisting of a flat grinding blades of diameter 50 mm. The grinder had 40 levels of grinding, 0 for the coarsest point level and 40 for the finest. The right degree of grinding was chosen in order to produce an espresso of 25 ml in 25–30 s. Therefore, a grinding trial was performed for each sample before performing the sensory evaluation. Final grind setting for Robusta samples ranged from 29 to 32, for Arabica ones from 23 to 26, and for the commercial samples around 28. Espresso coffees were brewed with a “Nuova Simonelli‐Musica” machine (Nuova Simonelli, Macerata, Italia). Fourteen grams of ground coffee were placed in a double portafilter, compacted with manual pressure, and extracted with water at 88–95°C and about 9 bar, obtaining two espresso coffees with an approximate volume of 25 ml each. Bottled natural mineral water was used for the extraction of coffees (Leggera, Gaudianello S.p.A., Italy).

### 2.2 Physicochemical analysis of coffee samples

#### 2.2.1 Moisture content

The moisture content (%) of coffee powder was determined according to the Association of Official Analytical Chemists method (AOAC, 1999). Twenty grams of coffee powder were placed on a previously weighted evaporating dish. Samples were dried at 105°C until constant weight. After cooling in desiccator, dishes with dried sample were weighted. Measurements were taken in triplicate.

#### 2.2.2 Bulk density

The bulk density of a powder is the mass of the powder divided by its bulk volume (g/cm^3^). Coffee samples were analyzed according to the Doğan et al. (2019) method. For this purpose, a 10 ml graduated cylinder was used and filled with coffee powder. The loose bulk density of sample corresponded to the weight of coffee powder (g) needed to fill the cylinder (volume expressed in cm^3^), without tapping the cylinder. Measurements were taken in triplicate.

#### 2.2.3 Solubility index

The solubility index was measured as reported by Doğan et al. (2019); four grams of coffee powder were mixed with 40 ml of distilled water at 80°C and continuously stirred at this temperature for 30 min. Then, the samples were centrifuged at 5500 rpm for 12 min. Approximately 15 g of supernatant were transferred in an evaporating dish and dried at 105°C until constant weight. Solubility index of the samples was calculated using the following formula:

$$\begin{array}{ccc} & & \mathrm{Solubility}\;\mathrm{index}\;\left(\%\right)=\\ & & \left(\mathrm{Weight}\;\mathrm{dried}\;\mathrm{supernatant}/\mathrm{Initial}\;\mathrm{weight}\;\mathrm{of}\;\mathrm{powder}\;\mathrm{coffee}\right)\\ & & *100 \end{array}$$

#### 2.2.4 Color determination

The color measurement of coffee powder was determined using a colorimeter (Konica Minolta CR‐300 Chroma Meter, Japan) and expressed by L*(lightness value), a* (green‐red component), and b* (blue‐yellow component) CIE Lab parameters. The last two parameters have been considered by a single index, called “chroma”. Standard 90‐mm‐diameter Petri dishes were filled with coffee powder and the measurements were taken from the top surface, in triplicate.

$$\mathrm{Chroma}=\surd a^{2}+b^{2}$$

#### 2.2.5 Total phenolic content

Total phenolic content (TPC) was determined according to Wongsa et al. (2019). The extraction step was carried out by mixing 1 g of coffee powder with 10 ml of hot distilled water at 90–95°C. The mixture was centrifuged at 2500 rpm for 20 min. For the determination of TPC, 1 ml of supernatant was mixed with 5 ml of 10% of Folin‐Ciocâlteu reagent (v/v) (Titolchimica, Italy) and, after an incubation period of 5 min, 4 ml of 7.5% sodium carbonate (w/v) (Sigma‐Aldrich, Milan, Italy) were added. The samples were incubated at room temperature for 60 min, in the dark. Subsequently, the absorbance was read at 765 nm. In order to determine the TPC, a standard curve was prepared by using both gallic acid (concentrations in the range 5–50 µg/ml) and chlorogenic acid (in the range 5–100 µg/ml), purchased from Sigma‐Aldrich. Results were expressed as mg gallic acid equivalent/g coffee powder (mgGA/g) and as mg chlorogenic acid equivalent/g coffee powder (mgCGA/g). Measurements were taken in triplicate.

#### 2.2.6 Caffeine content

The caffeine content was determined in ground coffee, prepared in accordance with the procedure described by Caprioli et al. (2014). In order to extract the caffeine from ground coffee, 1 g of sample and 4 g of magnesium oxide (Sigma‐Aldrich) were placed in a 100 ml flask, then ultrapure water at 90°C was added to capacity. For each sample, two replicates of extraction procedure were used. After shaking for 1 h at a constant temperature of 90°C, the mixtures were filtered through a 0.45 µm PTFE filter. Each sample extract was analyzed by HPLC in duplicate. Therefore, four caffeine measurements were available for each coffee sample. The extracted caffeine was quantified using a Varian Chromatography System 1220 Infinity LC comprising a 9012 pump and a 1260 Infinity II Diode Array Detector HS (Varian‐Agilent, Milan, Italy). HPLC‐UV/Vis analyses were performed at 272 nm. The injection volume was 20 µl and the HPLC operation mode was isocratic, at a flow rate of 1.0 ml/min. The mobile phase for HPLC analysis was water (A) containing 0.3% of formic acid and methanol (B) in the volume ratio 70:30. A Zorbax Eclipse Plus C18(4.6×150 mm, 5 µm) was used. Quantification of caffeine was performed using a four‐point (*n* = 2) external standard curve.

#### 2.2.7 Headspace (HS) evaluation by GC‐EIMS analyses

Each coffee sample (both coffee beans and ground beans) was put in a sealed glass crimp vial until analysis. As reported by Ascrizzi and Flamini (2020), for the headspace solid phase micro‐extraction (HS‐SPME), all the samples (5 g of coffee beans and 5 g of ground beans from each sample analyzed in triplicate) were individually put in 4 ml glass vials (up to one‐third of their total volume), which were then covered with aluminum foil and left to equilibrate at room temperature for 30 min. To avoid contamination, only one vial was opened each time and, instead of transfer of the contents (another possible source of contamination), the vial opening was immediately sealed with aluminum foil maintained in position with a rubber band. A solid phase micro‐extraction (SPME) device (Supelco, St. Louis, MO, USA) coated with DVB/CAR/PDMS (100 µm) was used for sampling, which was accomplished in an air‐conditioned room (22 ± 1°C) to guarantee a stable temperature. A 30 min sampling interval was experimentally determined to ensure the optimal fiber adsorption of the volatiles, avoiding over‐ and under‐saturation of the fiber. Once sampling was finished, the fiber was withdrawn into the needle and transferred to the injection port of the GC‐MS system. The desorption time was 10 min at 220°C. However, from previous analyses, it was noted that after 3 min, the fiber resulted clean. We used a longer time to further avoid possible contamination. Blanks were performed before each first SPME extraction, and randomly repeated during each series. Semi‐quantitative comparisons of relative peak areas were performed between the same compounds in different samples. Gas chromatography–electron impact mass spectrometry analyses were performed with an Agilent 7890B gas chromatograph equipped with an Agilent HP‐5MS capillary column (30 m × 0.25 mm; coating thickness 0.25 µm) and an Agilent 5977B single quadrupole mass detector (Agilent Technologies Inc., Santa Clara, CA, USA). The analytical conditions were as reported in Ascrizzi et al. (2017): injector and transfer line temperatures were 220 and 240°C, respectively; the oven temperature was programmed from 60 to 240°C at 3°C/min^1^; the used carrier gas was helium, at 1 mL/min; and the split ratio was 1:25. Acquisition parameters were as follows: full scan; scan range: 30–300 m/z; scan time: 1.0 s. The identification of constituents was based on a comparison of their retention times with those of authentic samples, and comparing their linear retention indices relative to a series of n‐hydrocarbons (C6–C25). Computer matching was also used against commercial (National Institute of Standards & Technology, 2014) and laboratory‐developed mass spectra library built up from pure substances and components of commercial essential oils of known composition and MS literature data (Adams, 2007).

### 2.3 Sensory analysis

#### 2.3.1 Sensory panel

A panel of 77 assessors, aged 20–60, was recruited to conduct a sensory analysis of coffee. The average age of the panelists was approximately 38, and median age was 40‐years‐old. The gender ratio was approximately 55% females and 45% males. All procedures performed in this study involving human participants were in accordance with the 1964 Helsinki Declaration and its later amendments. In addition, we followed Regulation (EU) 2016/679 of the European Parliament and of the Council of 27 April 2016 on the protection of natural persons regarding the processing of personal data and on the free movement of such data.

#### 2.3.2 Evaluation procedure

Sensory evaluation was performed following the approach described in Giacalone et al. (2019) according to which 83 consumers were first asked to rate overall liking of Arabica coffee samples (differing in roasting conditions) and then to complete a CATA questionnaire consisting of 30 attributes. In this study, sensory analysis was performed in individual booths under white light at room temperature in the Sensory Analysis Laboratory of the University of Basilicata, Italy, complying with the structural parameters set out in ISO guidelines (UNI‐ISO 8589:2010). The 20 coffee samples were analyzed in 5 sessions, just so that each consumer assessed 4 samples per session in order to minimize fatigue. In the first two sessions, Robusta samples were tested, Arabica samples in the third and four sessions and, in the last one, the four commercial blends were evaluated. Arabica and Robusta coffees have different sensory characteristics: large difference between two samples may mask small differences between others, leading to an increased perceived similarity among products thus affecting hedonic results. For this reason, in this sensory analysis, samples from different varieties were evaluated in different sessions, and not according to a fully randomized design, in order to avoid convergence effect. Coffee samples were brewed immediately before being served, in accordance with the preparation procedure previously described. Samples were served in brown plastic cups, identified with a three‐digit random code. The serving order of samples was randomized and balanced for each session using a Latin Square order designed by FIZZ software (BioSystem, France) so as to avoid carryover effects. Water was provided to cleanse the palate between samples, according to an appropriate washing procedure lasting for 90 s. For each sample, consumers were first asked to evaluate the overall liking and, then, complete the CATA test. Each session lasted about 30 min, on average. Sensory evaluation was conducted by using FIZZ software (BioSystem).

#### 2.3.3 Overall liking

Overall liking was evaluated using the Generalized Labelled Magnitude Scale (gLMS). It is a semantically labeled scale that enables hedonic measurements, thanks to verbal instructions on the scale (Lim et al., 2009). The scale ranges from 0: “most disliked sensation imaginable” to 100: “most liked sensation imaginable” (0: “il più sgradevole che si possa immaginare”; 100: “il più gradevole che si possa immaginare” are the corresponding terms used by panelists during the sensory evaluation).

#### 2.3.4 CATA questionnaire

CATA test was performed using a list of 18 attributes related to odor, taste, flavor, and mouthfeel sensations. CATA questionnaire included both sensory attributes and hedonic terms: these latter are preferably related to the vocabulary that consumers commonly used for describing the products. For this reason, CATA method used in this study cannot be reproduced precisely in the same way by other consumer populations. Attributes used in this study were as follows: strong (odor and taste sensations perceived at high intensity), delicate (odor and taste sensations perceived at low intensity), balanced (all odor and taste sensations well‐balanced perceived), long aftertaste (long residual taste sensation that persists for a long period after swallowing the product), tobacco (flavor associated with roasted tobacco), caramel (flavor associated with caramels), liquorice (flavor associated with liquorice), chocolate (flavor associated with 100% dark chocolate), nutty (flavor associated with roasted hazelnuts), roasted (flavor associated with products cooked to a high temperature by dry heat), burnt (flavor associated with over‐cooked or over‐roasted product), earthy (flavor associated with wet soil), grassy (flavor associated with fresh‐cut grass), citrus (flavor associated with lemon), bitter (basic taste produced by dilute aqueous solutions of caffeine), sweet (basic taste produced by dilute aqueous solutions of sucrose), acidic (basic taste produced by dilute aqueous solutions of citric acid), and astringent (tactile sensation, described as puckering or dry, produced by tannins). The terms were selected based on a previous consumer test (Giacalone et al., 2019). All attributes were presented in balanced and randomized order to avoid bias in the consumers’ responses. Consumers were asked to check all the terms that they considered appropriate to describe the sample under assessment.

### 2.4 Statistical analysis

Physicochemical analyses were performed in triplicate. The results were expressed as the mean of three values ± standard deviations. Two‐way analysis of variance (ANOVA) with interaction using variety (Arabica, Robusta) and geographical origin (Africa, America, Asia) as fixed factors was performed in order to compare replicates and detect the significant effect of factors on chemical composition. When ANOVA was significant at 95%, Tukey's HSD (Honestly Significantly Different) test was computed for post hoc analysis, in order to identify individual differences.

Overall liking scores were analyzed using one‐way analysis of variance (ANOVA) followed by Tukey's HSD test (*p* < 0.05) to determine whether explanatory variable (coffee samples) affected liking scores (dependent variable). Pearson correlation was computed to determine the associations between physicochemical attributes (chemical composition and volatile compounds groups) and overall liking.

CATA results were transformed in a binary table and then submitted to Cochran's *Q* test to identify differences between samples based on attributes chosen, followed by multiple pairwise comparisons using critical difference (Sheskin) procedure through “CATA analysis” tool in XLSTAT. CATA data were reported as frequency of chosen attributes and submitted to the correspondence analysis (CA). CA was conducted to visualize the relationship between samples and sensory attributes. Penalty lift analysis was carried out to evaluate the impact of each attribute on the liking score. All these statistical analyses were performed using the software XLSTAT Premium (Version 2020.3.1, Addinsoft, Paris, France).

For the head space volatile organic compounds (VOCs) analyses, the percentage of dissimilarity contribution of all the compounds in their complete compositions was evaluated using the similarity percentage test (SIMPER) with the Bray–Curtis distance/similarity measure with the PAST 3 Software. For all the 109 VOCs identified, accounting for at least 1.000% in the dissimilarity rate based on their form (whole or ground), a 3‐way ANOVA was performed to assess the statistical significance of the difference in their relative abundances, evaluating the effects of the variety, the geographical origin, the form (whole or ground), and all their combinations (Variety ˟ Origin, Variety ˟ Form, Origin ˟ Form, Form ˟ Variety, and Origin ˟ Form ˟ Variety). Hierarchical cluster (HC) and principal component (PC) analyses were performed with JMP 13.2.0 software (SAS Institute, Cary, NC, USA) considering the HSs complete compositions. The HSs HCA was conducted with the Ward's algorithm on normalized data, using Euclidean distances as a measure of similarity. To perform the PCA, linear regressions were operated on mean‐centered, normalized data of the covariance matrix to select the two highest principal components (PCs). This unsupervised method reduced the dimensionality of the multivariate data of the matrix, while preserving most of the variance (Ascrizzi et al., 2018). The chosen PC1 and PC2 studied 49.27% and 26.79% of the variance, respectively, for a total of 76.06% observed data.

## 3 RESULTS AND DISCUSSION

### 3.1 Physicochemical characteristics

The results for physicochemical analyses of all coffee samples parameters are presented in the supplementary material (Table S2). Results from this study showed the clear difference between Arabica and Robusta samples according to their physicochemical characteristics.

As demonstrated in Table 1, Arabica and Robusta were significantly different (*p* < 0.05) for each parameter taken into account. Robusta had higher value than Arabica for all the physicochemical attributes evaluated, except for moisture content. Caffeine content in Robusta samples was almost twice as much as in Arabica variety, as has been shown by other studies (Gaibor et al., 2020). As regard to sensorial characteristics, caffeine imparts bitterness and contributes to the distinctive flavor of the final beverage. Robusta variety is therefore used in combination with Arabica to strike the right balance between Robusta astringent and bitterness notes and Arabica acidic taste, but also to provide body and increase foam of coffee beverage, due to a higher dry matter content (Pereira et al., 2021). Phenolic compounds have a significant influence on sensorial characteristics of the final product, as well, imparting sourness, bitterness, and astringent notes in addition to their important antioxidant properties. Chlorogenic acids are the main phenolic compounds in coffee, but their content is influenced by raw material, roasting process, and brewing methods (Wongsa et al., 2019). In this study, the TPC was determined on ground coffee samples subjected to the same roasting level and hence the significant difference between Robusta and Arabica samples (*p* < 0.05) could be solely attributed to varietal differences.

**TABLE 1:** Differences between coffee samples according to their variety (Arabica and Robusta)

Physicochemical attributes	Arabica	Robusta
Moisture content (%)	1.73 ± 0.23 ^a^	1.59 ± 0.10 ^b^
Bulk density (g/cm^3^)	0.25 ± 0.01 ^b^	0.27 ± 0.01 ^a^
Solubility index (%)	10.66 ± 0.33 ^b^	11.05 ± 0.24 ^a^
L*	22.40 ± 1.67 ^b^	26.10 ± 1.82 ^a^
Chroma	22.59 ± 2.29 ^b^	26.04 ± 0.94 ^a^
Total phenolic content (mgGA/g)	1.93 ± 0.57 ^b^	2.52 ± 0.60 ^a^
Total phenolic content (mgCGA/g)	4.42 ± 1.30 ^b^	5.75 ± 1.37 ^a^
Caffeine content (mg/g)	14.52 ± 2.10 ^b^	24.77 ± 1.80 ^a^

mgGA/g = mg gallic acid equivalent/g coffee powder.

mgCGA/g = mg chlorogenic acid equivalent/g coffee powder.

Data are expressed as mean ± standard deviation. Values in the same row followed by a different letter are statistically different (*p* < 0.05), following pairwise comparison by Tukey's HSD test.

Coffee samples were also divided according to the continent of cultivation, in order to verify if the geographical origin affects physicochemical characteristics. Given the fact that no Robusta sample belonged to American origin, only Arabica samples were taken into account for this purpose in order to avoid unbalanced data. As reported in Table 2, coffee samples from America were statistically different from those deriving from Africa in moisture content, whereas they differed from Asian samples (*p* < 0.05) according to their caffeine content. A complementary second analysis to evaluate the effect of geographical origin on physicochemical attributes of coffee samples was performed taking into account all African and Asian coffees, leaving out American ones. In this case, there was a significant difference (*p* < 0.05) between Asian and African coffees only as regards moisture content, in particular coffee samples that belonged to Asia origin demonstrated a significantly higher moisture content than African coffees.

**TABLE 2:** Difference between Arabica coffee samples according to their geographical origin (Africa, America, Asia)

Physicochemical attributes	Africa	America	Asia
Moisture content (%)	1.53 ± 0.01 ^b^	1.86 ± 0.21 ^a^	1.70 ± 0.19 ^ab^
Bulk density (g/cm^3^)	0.25 ± 0.004 ^a^	0.25 ± 0.01 ^a^	0.25 ± 0.02 ^a^
Solubility index (%)	10.59 ± 0.21 ^a^	10.60 ± 0.28 ^a^	10.78 ± 0.39 ^a^
L*	21.87 ± 0.89 ^a^	22.66 ± 1.72 ^a^	22.40 ± 1.82 ^a^
Chroma	21.28 ± 2.11 ^a^	22.62 ± 2.01 ^a^	23.41 ± 2.24 ^a^
Total phenolic content (mgGA/g)	2.16 ± 0.22 ^a^	1.85 ± 0.59 ^a^	1.89 ± 0.62 ^a^
Total phenolic content (mgCGA/g)	4.93 ± 0.49 ^a^	4.22 ± 1.35 ^a^	4.33 ± 1.42 ^a^
Caffeine content (mg/g)	13.67 ± 0.62 ^b^	13.15 ± 0.45 ^b^	16.92 ± 1.91 ^a^

mgGA/g = mg gallic acid equivalent/g coffee powder.

mgCGA/g = mg chlorogenic acid equivalent/g coffee powder.

Data are expressed as mean ± standard deviation. Values in the same row followed by a different letter are statistically different (*p* < 0.05), following pairwise comparison by Tukey's HSD test.

No significant differences (*p* > 0.05) resulted in terms of TPC between coffee samples from different geographical origin, as also confirmed by Demianová et al. (2021). Qualitative analyses on phenolic compounds could be more appropriate to discriminate coffee samples according to their geographical origin. In example, Alonso‐Salces et al. (2009) reported that chlorogenic acid and cinnamoyl‐amino acid conjugate were useful for a more precise geographical identification of Robusta samples, whereas fatty acids, chlorogenic acids, and specific compounds were considered more appropriate indicators to assign geographical origin to Arabica coffees.

Two‐way ANOVA demonstrated that interaction between factors “variety” and “geographical origin” was not significant for each physicochemical parameter considered (*p* > 0.05).

### 3.2 Headspace compositions and statistical analyses

Among all analyzed headspaces (HSs), 109 VOCs were identified in total; all sample compositions are reported in Tables S3 to S6 of the Supplementary Material. The list of VOCs accounting for at least 1.000% in the dissimilarity rate among all samples is provided in Table 3.

**TABLE 3:** Compounds accounting for at least 1.000% in the dissimilarity rate according to the similarity percentage (SIMPER) test of the complete headspace compositions of all analyzed coffee samples; three‐way ANOVA of the compounds selected with the SIMPER test taking into account their i) variety (Arabica, Robusta, Blend), ii) geographical origin (Africa, America, Asia, Commercial), iii) form (whole, W; ground, G), and their full factorial combinations (the “Form ˟ Variety” combinations resulted not significant for all compounds, so it was not reported in the table)

**Compound**	**Individual % contribution**	**Cumulative %**	**Variety**	**Origin**	**Form**	**Variety ˟ Origin**	**Variety ˟ Form**	**Origin ˟ Form**	**Origin ˟ Form ˟ Variety**
Pyridine	7.773	7.773	* Arabica, *** Blend	* Asia	***		* (Arabica ˟ G)		
furfuryl alcohol	5.768	13.54	*** Arabica					* (Asia ˟ G)	
acetic acid	4.957	18.5	*** Arabica, ** Blend		***			* (Asia ˟ G)	* (Africa ˟ G ˟ Arabica)
*p*‐vinylguaiacol	4.951	23.45	*** Blend	** Africa, * Asia					* (Africa ˟ G ˟ Arabica)
2‐furfuryl acetate	4.79	28.24	*** Arabica, ** Blend	** Asia			* (Arabica ˟ G)	* (Asia ˟ G)	* (Africa ˟ G ˟ Arabica)
2,6‐diethylpyrazine	3.755	31.99	*** Arabica and Blend	*Africa, ** Asia					
2,6‐dimethylpyrazine	3.553	35.55	*Arabica	*Asia					
2‐methylpyrazine	3.207	38.75	** Arabica and Blend		***		* (Blend ˟ G)	*(Asia ˟ G)	*(Africa ˟ G ˟ Arabica)
2‐ethyl‐6‐methylpyrazine	2.775	41.53	*** Arabica	** Asia					
maltol	2.555	44.08	*** Arabica and Blend	*** Asia	**	*(Arabica ˟ Africa)			
5‐methylfurfural	2.149	46.23	*** Arabica, **Blend	** Asia	*			*(Asia ˟ G)	
trimethylpyrazine	2.102	48.34	*** Arabica	** Africa, *** Asia	*			*(Africa ˟ G)	
*p*‐ethylguaiacol	1.954	50.29	*Blend	*Africa, ** Asia		** (Arabica ˟ Africa)			
3‐methoxypyridine	1.903	52.19			**				
γ‐butyrolactone	1.878	54.07	*Blend				*(Arabica ˟ G)	** (Asia ˟ G)	*(Africa ˟ G ˟ Arabica)
2,3‐diethylpyrazine	1.795	55.87	*** Arabica, ** Blend	** Asia	*		** (Arabica ˟ G)		
2‐ethyl‐3,5‐dimethyl pyrazine	1.708	57.58	*** Blend	*Asia					
2‐furfurylfuran	1.532	59.11	*** Blend						
2‐ethylpyrazine	1.526	60.63	*** Arabica, ** Blend	*Africa	**		** (Blend ˟ G)	** (Asia ˟ G)	** (Africa ˟ G ˟ Arabica)
acetoxyacetone	1.507	62.14	** Arabica. *Blend	** Asia					
2‐ethyl‐5‐methylpyrazine	1.497	63.64	*** Arabica	** Africa and Asia					
2‐ethyl‐3‐methylpyrazine	1.348	64.99	*** Arabica	*Africa, ** Asia					
furfural	1.224	66.21	** Arabica	** Asia	**				
dihydro‐2‐methyl‐3(2H)‐furanone	1.121	67.33		**Asia					
3,5‐dimethylisoxazole	1.105	68.44	*** Arabica and Blend				** (Blend ˟ G)		
2‐acetylpyrrole	1.025	69.46			***		* (Blend ˟ G)	* (Asia ˟ G)	
2‐amino‐6‐methylpyridine	1.019	70.48	** Arabica	*Africa	***		** (Blend ˟ G)	* (Africa ˟ G)	* (Africa ˟ G ˟ Arabica)
*ortho*‐guaiacol	1.018	71.5	*Arabica	*** Africa	*		* (Arabica ˟ G)		

* *p* < 0.05.

** *p* < 0.01.

*** *p* < 0.001.

Ninety compounds in total were identified for Arabica samples (Tables S3 and S4, supplementary material). Pyrazines and furans were detected as the two‐most abundant chemical classes of VOCs in all Arabica samples; combined, they always accounted for over 50% of the total HS composition. Two Indian samples (A7 and A8) exhibited the highest pyrazine relative content, while furans were more represented in two South American (A3, Peruvian; A4, Brazilian) and one Indonesian (A9) samples. Among pyrazines, 2,6‐dimethyl pyrazine was the most abundant, as already reported for other monovarietal Arabica coffee HSs (Ascrizzi & Flamini, 2020), its aroma contribution is reported as sweet and nut‐like (Burdock, 2016; Flament, 2002). Other quantitatively relevant VOCs of this chemical group were 2‐methyl, 2‐ethyl‐6‐methyl, and 2,6‐diethyl pyrazine. 2‐Methyl pyrazine confers a desirable nutty and chocolate‐like aroma, and a pleasantly roasted (The Good Scents Company, 2020) and hazelnut‐like odor contribution is described for 2,6‐diethyl pyrazine (Id & Flavic, 2006). Furfuryl alcohol, 2‐furfuryl acetate, and 5‐methyl furfural were detected as the most abundant furans in all Arabica HSs. 5‐Methyl furfural is a desirable VOC in coffee HS, as it confers spicy, sweet, and caramel‐like notes (Arctander, 1969; Burdock, 2016). Pyridine was detected as the most abundant compound of the third‐most represented HS chemical class, constituted by pyridines and piperidines. Although it is commonly described as an off‐flavor, its dilution in coffee confers favorably warm and smoked notes (Arctander, 1969) and it is also reported in other high‐quality mono‐varietal Arabica coffee HSs (Ascrizzi & Flamini, 2020).

A total of 88 compounds were detected among all Robusta samples (Table S5, supplementary material). Pyrazines were the most abundant chemical group, accounting for relative abundances ranging from a minimum of 33.4% to a maximum of 57.3%. As in the Arabica samples, 2,6‐dimethyl pyrazine was the most abundant of this group. 2‐Ethyl‐6‐methyl pyrazine followed; its “green” and roasted notes are reported as pleasant aroma contributions (Afoakwa, 2016). 2‐Methyl and trimethyl pyrazine also exhibited relevant relative concentrations; for the latter, an earthy, cocoa‐ and peanut‐like aroma character is reported (Bonvehí, 2005; Frauendorfer & Schieberle, 2008). Furans were detected as the second‐most represented chemical class of VOCs in Robusta HSs, the most abundant of which were the same compounds listed above for the Arabica samples. Differently from the latter, however, phenols followed as the third‐most represented VOC group, among which *p*‐vinyl, *p*‐ethyl, and *o*‐guaiacol exhibited the highest relative abundances in all Robusta samples. All these phenols share spicy, vanilla‐like, and woody aroma notes (The Good Scents Company, 2021a, 2021b, 2021c).

These findings are in accordance with the study of Colzi et al. (2017), which reported an overall predominance of furans in Arabica samples compared to Robusta, whereas the opposite was true for phenols (guaiacol derivatives).

For the commercial blend samples (Table S6, supplementary material), 91 compounds were identified among all the analyzed HSs. As emerged for the Arabica samples, pyrazines, furans, and pyridines were the most represented chemical groups, of which the most abundant compounds were the same already listed for the Arabica coffees.

Among all the factors taken into account, the varietal one induced the highest degree of variability between the samples for almost all the compounds selected by the SIMPER analysis (Table 3), followed by the geographical origin and finally by the form (whole or ground beans). The interactions between the analyzed factors evidenced lower degrees of significance on the HSs compositions. As regards the effect of variety, the most significant (*p* < 0.001) quantitative differences between the samples were detected for all the selected pyrazines (with the exception of 2,6‐dimethyl pyrazine) and furans (with the exception of furfural), whose relative presence was significantly different among the Arabica coffees and/or the commercial blends. The evidenced higher compositional differentiation of the Arabica samples compared to the Robusta coffee is in accordance with Freitas et al. (2001). The origin factor followed; it induced the most significant (*p* < 0.001) differences between the Asian and African samples for maltol and ortho‐guaiacol, respectively. Overall, Asian and African coffees were characterized by a higher degree of difference among the samples of the same geographical origin for the majority of the SIMPER‐selected compounds, which might probably be explained by the higher differences among the environmental habitats in these two continents compared to the South American one. Unsurprisingly, the form (whole or ground) factor induced the most significant differences (*p* < 0.001) in the emission of low molecular weight compounds, such as acetic acid, pyridine, and 2‐methyl pyrazine. In addition, even if the grinding process is influenced by several factors, including the variety (Seninde & Chambers, 2020), in this study “Form ˟ Variety” combinations resulted not significant for all compounds, as shown in Table 3.

**FIGURE 1:**
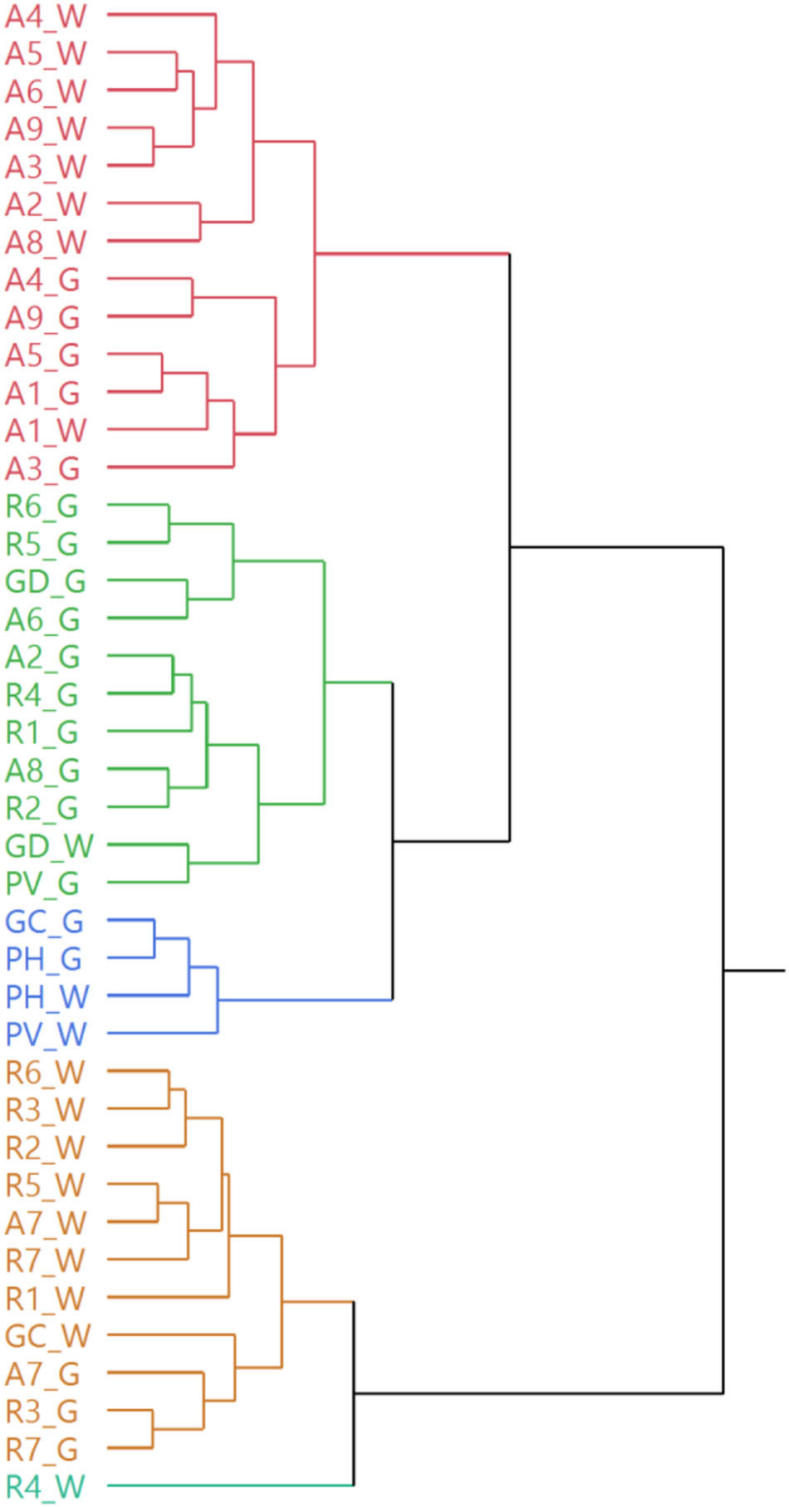
Dendrogram of the hierarchical cluster analysis (HCA) performed on the complete headspace compositions of all the analyzed samples, both in their whole (W) and ground (G) forms

The hierarchical cluster analysis (HCA) performed on the complete HS compositions of all the analyzed samples (Figure 1) distinguished two macro‐clusters. The first one (Cluster1) included a subcluster homogenously composed of Arabica samples, another one (Cluster3) of Blend ones whereas the third subcluster (Cluster2) was more heterogeneously composed by Robusta coffees (mainly), some Arabica ones, and the GD Blend sample. Cluster2 was also mainly composed of ground samples. The second macrocluster was composed of an almost completely homogeneous subcluster (Cluster4), comprising mostly Robusta whole samples, and one subcluster (Cluster5) composed of only one Robusta coffee. With few exceptions, the dendrogram evidenced a quite sharp separation of the samples based on their variety (Arabica, Robusta, or Blend) according to the volatile composition of sample HSs.

**FIGURE 2:**
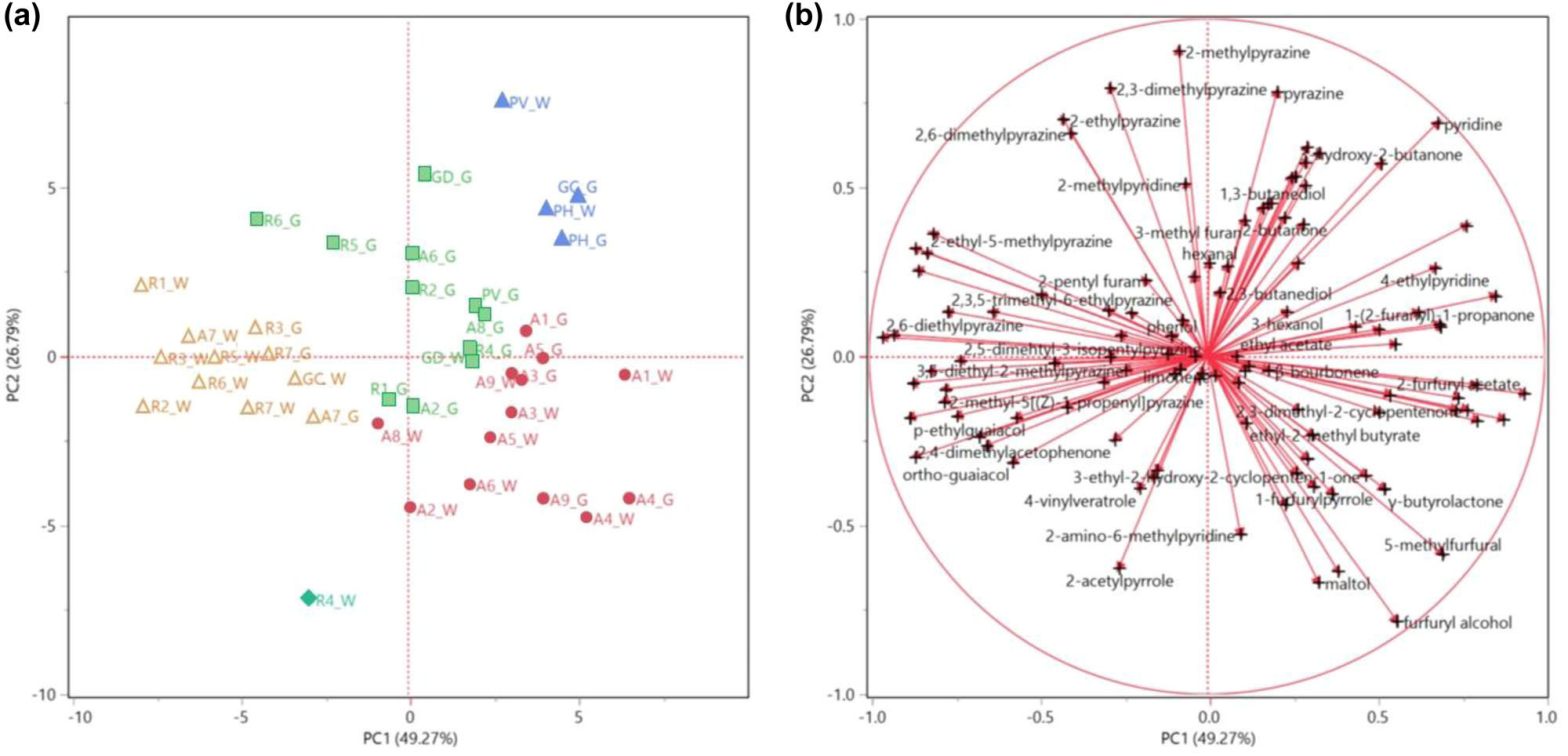
Score (a) and loading (b) plots of the principal component analysis (PCA) performed on the complete headspace compositions of all the analyzed samples both in their whole (W) and ground (G) forms.

This behavior was further confirmed by the principal component analysis (PCA), as shown in the obtained score plot (Figure 2a)). Cluster1 composed of Arabica samples only was plotted on the right quadrants (PC1 > 0), mostly on the bottom one (PC2 < 0). This positioning was mostly driven by the furfuryl alcohol, 5‐methylfurfural, and maltol vectors, which pointed toward the bottom right corner of the plot (Figure 2b)), whose relative concentration was overall higher in the Arabica samples compared to the Robusta ones. Cluster4, comprising mostly Robusta samples, was, instead, positioned on the left quadrants (PC1 < 0), as well as the Robusta sample 4, constituting a subcluster of its own in the HCA dendrogram (Figure 1). Both ortho‐ and *p*‐vinyl‐ guaiacol vectors, of which these samples exhibited a higher relative content, lay in this quadrant (Figure 2b). Cluster2, more heterogeneously composed, was plotted between Cluster1 and Cluster4, toward the central part of the plot. With the exception of the Blend sample GC in its whole form, all the Blend coffees were plotted in the upper right quadrant (PC1 and PC2 > 0), more oriented toward the Arabica samples, thus confirming their higher compositional similarity. This positioning of the Blend coffees was mainly due to their overall higher relative abundance, compared to the other samples, of pyridine, whose vector lays in this quadrant (Figure 2b).

The HS compositions analyzed by multivariate statistical analysis were, thus, able to quite sharply discriminate the samples based on their variety.

### 3.3 Sensory evaluation

#### 3.3.1 Overall liking

Overall liking mean scores are reported in Table S7. Robusta samples were rated significantly higher than Arabica and Blend ones (*p* < 0.05). These findings are in contrast with previous results reported in the literature (Kim et al., 2016), according to which Arabica samples received higher liking score than Robusta ones. However, only South‐Korean female consumers were recruited in Kim et al. (2016), aged between 18 and 36 years. Therefore, the different results could be due to different panel specifics. As regards the influence of geographical origin, no statistical differences between coffee samples from different origins were observed in terms of overall liking (*p* > 0.05).

In general, coffee samples were rated as “dislike moderately” to “neutral”: coffee blend sample GD (composed of 50% Robusta and 50% Arabica) ranked the highest in terms of overall liking whereas coffee sample A9 scored the lowest.

Generally, correlations between relative abundance of volatile compound groups (%) and overall liking confirmed the role of aromatic profiles in driving coffee liking (Mahmud et al., 2020). A significant (*p* < 0.05) negative influence of acids and aldehydes on overall liking of coffee samples emerged (*r* = −0.668 and *r* = −0.742, respectively), giving the beverage an acidic and a grassy‐like flavor. On the other hand, pyrazines were positively related to overall liking (*r* = 0.619; *p* < 0.05), suggesting that roasted and nutty attributes, associated to pyrazines content, could be acceptable sensory notes to be attributed to the coffee beverage. This was also confirmed by CATA test results, described in section 3.3.2.

#### 3.3.2 CATA results

Cochran's *Q* test allowed identifying differences between coffees according to frequency with which sensory attributes were selected for evaluation of sample (Tab. S8). Samples resulted statistically different (*p* < 0.05) only for 3 out of 18 sensory attributes of CATA test, which were: grassy, citrus, and acidic. Grassy and acidic were associated with a higher frequency to Arabica samples than to Robusta ones as a result of the significant higher relative abundance (%) of aldehydes and acids in Arabica samples compared to Robusta (*p* < 0.05). The panel was, thus, able to sensorially perceive the different volatile profiles of the two coffee varieties. Moreover, it was expected that Robusta samples, with a significantly higher caffeine content than Arabica ones, would have been considered more bitter than Arabica. Contrary to expectations, results from this study showed no significant difference between Arabica and Robusta samples as regards the attribute bitter, probably because caffeine is not the sole responsible factor for the bitterness of coffee, but that other compounds are also involved (Gao et al., 2021). In addition, bitterness is highly influenced by physiological attributes of the consumer, such as fungiform papillae density, response to the bitter taste (PROP status), and rate of caffeine metabolism (Spinelli et al., 2017). Thus, the choice of bitter attribute may depend not only on the physicochemical characteristics of coffee, but also on individual characteristics of consumer.

**FIGURE 3:**
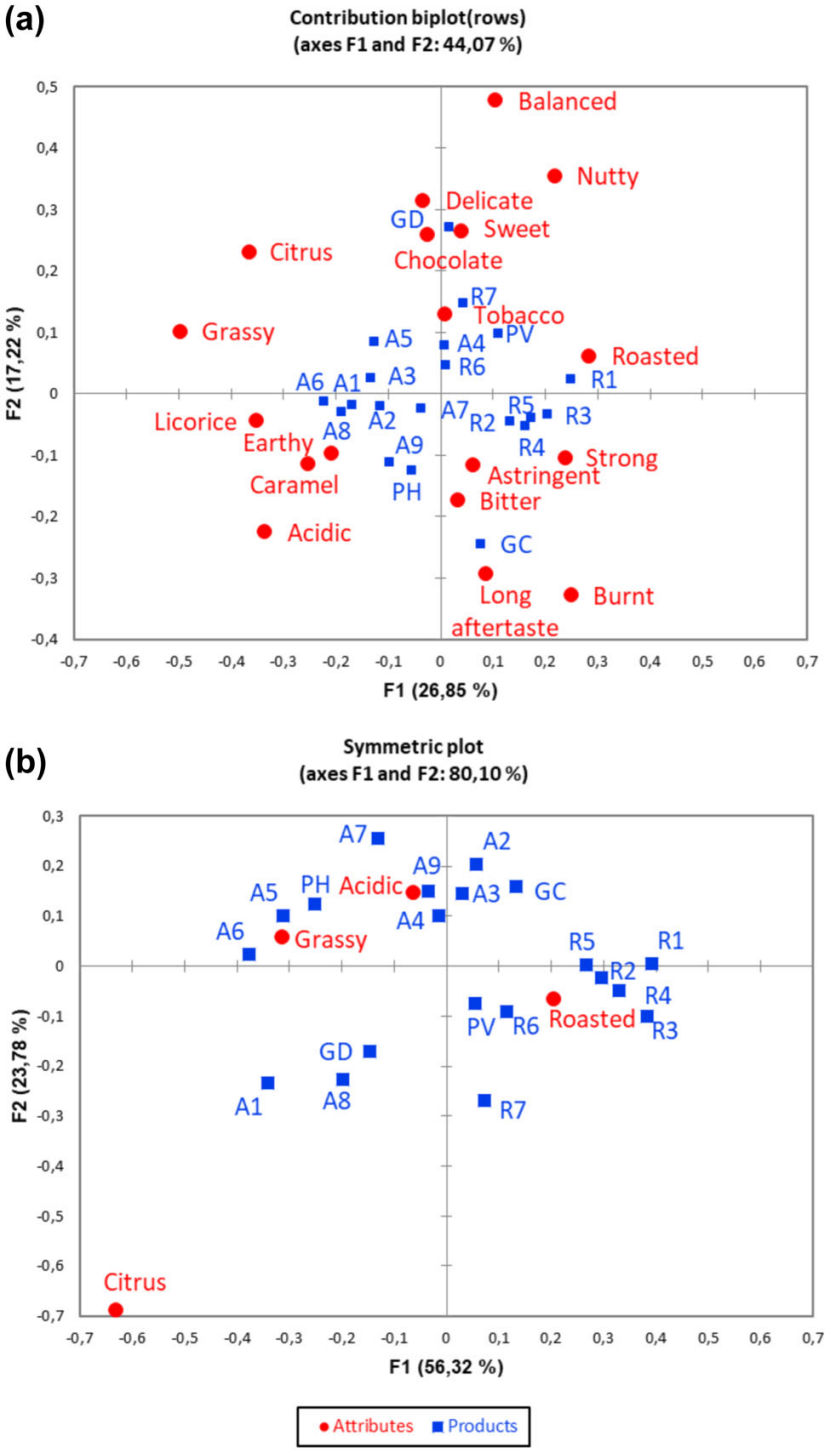
Correspondence analysis bi‐plot using all of the 18 CATA terms (a) and correspondence analysis bi‐plot using CATA terms selected according to Cochran's *Q* test results (b)

The most preferred coffee sample (GD) was considered the most balanced and the less acidic. Correspondence analysis (CA) bi‐plot using all 18 sensory attributes (Figure. 3a) suggested that Robusta samples were characterized by a roasted, strong, astringent, and bitter sensory profile, whereas Arabica ones were associated to attributes such as earthy, caramel, liquorice, and tobacco. Astringency, acidity, and bitterness of coffee arise from chlorogenic acids content, whereas pyrazine, pyrroles, and pyridines are responsible of roasted sensory attribute (Seninde & Chambers, 2020). As previously stated, Robusta demonstrated a TPC, expressed as chlorogenic acid and pyrazines content higher than Arabica samples (*p* < 0.05), proving how the chemical composition of ground coffee affects the sensory perception of coffee beverage. Chocolate, sweet, delicate, and balanced were highly related to GD, the most preferred sample. These results confirm previous findings in literature (Giacalone et al., 2019) showing that the production of a coffee characterized by well‐balanced intensity of sensory attributes, with no predominance of one attribute over the other, is important for high consumer satisfaction. A significant (*p* < 0.05) positive correlation between overall liking and the chosen frequency of delicate and balanced (*r* = 0.457 and *r* = 0.712, respectively) was detected suggesting that they could be positive drivers of coffee liking. However, these results could be not sufficient to explain the real sensory profile of coffee samples because CA of the 18 CATA terms explained low total variance. Following the approach described in Heo et al. (2019), performing a CA using only attributes with significant difference (*p* < 0.05) in the citation frequency, according to Cochran's *Q* test results, the variability explained increased from 44.07% to 80.10%. Robusta samples were positioned in quadrant 4(Figure 3b), mainly considered as roasted. A significant positive correlation (*r* = 0.517; *p* < 0.05) was observed between the frequency of use of the roasted attribute and the overall liking score, suggesting it could be considered a positive sensory driver for the acceptability of coffee. Arabica coffees were grouped in quadrant 1 and 2, described primarily by acidic and grassy attributes, both negatively correlated with overall liking (*r* = −0.469, *p* < 0.05 and *r* = −0.324, *p* > 0.05). A1 and A8 samples deviated from the other Arabica coffees having a sensory profile more similar to the sample GD. CATA test proved to be an efficient method, alternative to descriptive analysis, in providing both information about consumer perception of coffee samples and suitable sensory profile of coffee beverages.

Further confirmation derived from penalty lift analysis, a useful approach to identify drivers of liking and disliking by exploring the impact of sensory attributes on hedonic scores (Di Cairano et al., 2021; Meyners, 2016). Roasted, related to the presence of pyrazines, was the only attribute with a significant impact on liking score (*p* < 0.0001), implying a mean increase of 4.9 liking points. Attributes such as grassy, citrus, and acidic, associated to aldehydes and acids, respectively, reduced the liking score of 3.8, 4.3 and 12.5, respectively, but their impact was not significant.

## 4 CONCLUSION

Sensory analysis tests, aiming to investigate consumers’ preference, are useful tools for optimization of food processes or for the development of a new food product, which could be successful in the market. For coffee manufacturers, it is still more important to explore the main factors influencing consumers’ acceptability since coffee is a widespread and highly consumed beverage. Unfortunately, several elements with a pivotal impact on quality of final beverage must be taken into account, making its optimization a difficult and complex practice. It is well known that the coffee variety affects the chemical composition of coffee bean, as also confirmed by the significant differences between physicochemical parameters of coffee samples analyzed in this study. In addition, a significant influence of the variety on overall liking of coffee beverage was detected in this work: Robusta coffees obtained a higher liking score than Arabica ones (*p* < 0.05). No significant differences between samples, in terms of physicochemical attributes and overall liking score, were detected according to their geographical origin. Coffee has a rich and varied aromatic profile. It depends especially on roasting and brewing conditions but also on the number of chemical compounds that are precursors of specific odor/flavor attributes to be found in the raw starting material. From our results, volatile compounds groups such as acids, aldehydes, and pyrazines were the main precursors of sensory attributes showing a significant mean impact on liking score of mono‐variety and mono‐origin coffees, according to CATA results. Therefore, CATA method and penalty lift analysis confirmed the relationship between physicochemical parameters, mainly volatile compounds, and overall liking of coffee samples. In conclusion, the sensory attribute roasted (related to pyrazines amount) was well accepted by consumers; on the other hand, acidic and grassy sensory attributes (associated with the presence of acids and aldehydes, respectively) were considered as negative drivers of coffee liking.

## AUTHOR CONTRIBUTIONS

**Nicola Condelli**: Conceptualization; Formal analysis; Project administration; Visualization; Writing – original draft. **Nazarena Cela**: Conceptualization; Formal analysis; Investigation; Visualization; Writing – original draft. **Teresa Scarpa**: Formal analysis; Resources; Writing – review & editing. **Luigi Milella**: Conceptualization; Supervision; Visualization. **Roberta Ascrizzi**: Conceptualization; Formal analysis; Investigation; Visualization; Writing – original draft. **Guido Flamini**: Conceptualization; Supervision; Visualization. **Fernanda Galgano**: Project administration; Supervision; Visualization; Writing – review & editing

## CONFLICT OF INTEREST

The authors declare no conflict of interest.

## Supporting information


**Table S1**. Composition and information of coffee samples.
**Table S2**. Mean value ± standard deviation of physicochemical attributes
**Table S3**. Complete headspace compositions of the whole (W) and ground (G) Arabica samples (A1 to A4).
**Table S4**. Complete headspace compositions of the whole (W) and ground (G) Arabica samples (A to A4).
**Table S5**. Complete headspace compositions of the whole (W) and ground (G) Robusta samples.
**Table S6**. Complete headspace compositions of the whole (W) and ground (G) commercial blend samples.
**Table S7**. Mean liking scores of coffee samples
**Table S8**. Sensory attributes with significantly difference according to citation frequencies (%).
