# Supplementary material for: Drivers of coffee liking: Effect of physicochemical characteristics and aromatic profile on consumers’ acceptability of mono‐origin and mono‐variety coffees
Source: J Food Sci. 2022 Sep 16;87(10):4688–702. doi: 10.1111/1750-3841.16323 (PMC9826037; doi:10.1111/1750-3841.16323)
Supplement: Supplementary file 1 — Table S1. Composition and information of coffee samples. Table S2. Mean value ± standard deviation of physicochemical attributes Table S3. Complete headspace compositions of the whole (W) and ground (G) Arabica samples (A1 to A4). Table S4. Complete headspace compositions of the whole (W) and ground (G) Arabica samples (A to A4). Table S5. Complete headspace compositions of the whole (W) and ground (G) Robusta samples. Table S6. Complete headspace compositions of the whole (W) and ground (G) commercial blend samples. Table S7. Mean liking scores of coffee samples Table S8. Sensory attributes with significantly difference according to citation frequencies (%). [file JFDS-87-4688-s001.docx]

**Supplementary material**

**Table S1.** Composition and information of coffee samples.

| **Sample ID** | **Geographical Origin** | | **Variety** |
| --- | --- | --- | --- |
|  | **Continent** | **Country** |  |
| A1 | Africa | Kenya | Arabica |
| A2 | Africa | Congo | Arabica |
| A3 | America | Peru | Arabica |
| A4 | America | Brazil | Arabica |
| A5 | America | Costarica | Arabica |
| A6 | America | Nicaragua | Arabica |
| A7 | Asia | Flores | Arabica |
| A8 | Asia | India | Arabica |
| A9 | Asia | Indonesia | Arabica |
| GC | Commercial | Commercial | Blend |
| GD | Commercial | Commercial | Blend |
| PH | Commercial | Commercial | Blend |
| PV | Commercial | Commercial | Blend |
| R1 | Africa | Congo | Robusta |
| R2 | Asia | Vietnam | Robusta |
| R3 | Asia | India | Robusta |
| R4 | Africa | Tanzania | Robusta |
| R5 | Africa | Ivory Coast | Robusta |
| R6 | Asia | Cherry | Robusta |
| R7 | Africa | Uganda | Robusta |

**Table S2.** Mean value ± standard deviation of physicochemical attributes

| **Samples ID** | **Moisture content (%)** | **Bulk Density (g/cm³)** | **Solubility Index  (%)** | **Colour** | | **Total Phenolic Content** | | **Caffeine content (mg/g of powder)** |
| --- | --- | --- | --- | --- | --- | --- | --- | --- |
|  |  |  |  | **L*** | **Chroma** | **mgGA/g** | **mgCGA/g** |  |
| A1 | 1.44±0.05^j^ | 0.25±0.002 ^hij^ | 10.39±0.09 ^i^ | 21.16±0.83 ^j^ | 19.18±0.20 ^j^ | 2.34±0.14 ^bcd^ | 5.36±0.32 ^bcd^ | 14.13±0.46 ^g^ |
| A2 | 1.62±0.06^fghi^ | 0.25±0.007 ^ghij^ | 10.79±0.11 ^efg^ | 22.58±0.40 ^hi^ | 23.38±0.16 ^e^ | 1.97±0.12 ^cde^ | 4.50±0.27 ^cde^ | 13.04±0.09 ^hi^ |
| A3 | 1.59±0.02^ghi^ | 0.25±0.015 ^fgh^ | 10.71±0.12 ^gh^ | 21.89±0.30 ^hij^ | 20.81±0.05 ^h^ | 1.48±0.04 ^efg^ | 3.39±0.08 ^efg^ | 13.09±0.29 ^hi^ |
| A4 | 1.75±0.08^cdef^ | 0.25±0.003 ^fghi^ | 10.40±0.04 ^i^ | 21.54±0.54 ^ij^ | 20.58±0.49 ^hi^ | 1.11±0.02 ^g^ | 2.53±0.06 ^g^ | 12.51±0.10 ^i^ |
| A5 | 2.05±0.09 ^a^ | 0.26±0.005 ^efg^ | 10.82±0.51 ^efg^ | 25.40±0.78 ^de^ | 25.26±0.22 ^cd^ | 2.38±0.35 ^bcd^ | 5.45±0.81 ^bcd^ | 13.16±0.12 ^hi^ |
| A6 | 2.03±0.14 ^a^ | 0.24±0.008 ^j^ | 10.46±0.06 ^hi^ | 21.79±1.29 ^hij^ | 23.84±0.18 ^e^ | 2.42±0.17 ^bc^ | 5.53±0.39 ^bc^ | 13.73±0.03 ^gh^ |
| A7 | 1.77±0.09 ^cd^ | 0.28±0.004 ^b^ | 11.24±0.09 ^bc^ | 24.37±0.42 ^ef^ | 25.25±0.21 ^cd^ | 2.62±0.33 ^ab^ | 6.00±0.75 ^ab^ | 19.39±0.52 ^e^ |
| A8 | 1.88±0.07 ^bc^ | 0.24±0.005 ^j^ | 10.38±0.30 ^i^ | 22.77±0.30 ^gh^ | 24.73±0.13 ^d^ | 1.32±0.54 ^fg^ | 3.03±1.24 ^fg^ | 15.33±0.23 ^f^ |
| A9 | 1.45±0.06 ^j^ | 0.24±0.007 ^ij^ | 10.73±0.21 ^fgh^ | 20.05±0.57 ^k^ | 20.27±0.25 ^hi^ | 1.73±0.15 ^ef^ | 3.97±0.35 ^ef^ | 15.89±1.17 ^f^ |
| GC | 1.75±0.16 ^cde^ | 0.30±0.003 ^a^ | 11.46±0.03 ^ab^ | 23.71±0.25 ^fg^ | 22.44±0.28 ^f^ | 2.65±0.81 ^ab^ | 6.05±1.84 ^ab^ | 26.31±0.03 ^a^ |
| GD | 1.52±0.10 ^hij^ | 0.31±0.002 ^a^ | 10.36±0.18 ^i^ | 22.56±0.37 ^hi^ | 22.54±0.31 ^f^ | 1.81±0.07 ^def^ | 4.13±0.17 ^def^ | 19.39±0.23 ^e^ |
| PH | 1.94±0.12 ^ab^ | 0.26±0.010 ^ef^ | 11.02±0.11 ^cdef^ | 21.27±0.73 ^j^ | 20.07±0.26 ^i^ | 1.29±0.27 ^fg^ | 2.96±0.61 ^fg^ | 15.30±0.20 ^f^ |
| PV | 1.55±0.02 ^ghij^ | 0.26±0.010 ^efgh^ | 11.56±0.08 ^a^ | 22.17±0.26 ^hij^ | 21.67±0.25 ^g^ | 1.32±0.80 ^fg^ | 3.01±1.83 ^fg^ | 19.49±1.10 ^e^ |
| R1 | 1.49±0.05 ^ij^ | 0.30±0.004 ^a^ | 10.81±0.24 ^efg^ | 27.70±0.30 ^ab^ | 26.07±0.13 ^b^ | 3.18±0.21 ^a^ | 7.26±0.49 ^a^ | 24.65±0.45 ^b^ |
| R2 | 1.53±0.02 ^ghij^ | 0.27±0.009 ^cde^ | 11.13±0.17 cd | 28.48±0.17 ^a^ | 26.95±0.15 ^a^ | 1.91±0.03 ^cde^ | 4.37±0.08 ^cde^ | 25.24±0.42 ^b^ |
| R3 | 1.65±0.03 ^defg^ | 0.28±0.001 ^bc^ | 10.90±0.08 ^defg^ | 26.39±0.20 ^cd^ | 27.44±0.08 ^a^ | 2.68±0.28 ^ab^ | 6.13±0.63 ^ab^ | 25.31±0.73 ^b^ |
| R4 | 1.51±0.08 ^hij^ | 0.26±0.005 ^ef^ | 11.44±0.14 ^ab^ | 24.79±0.33 ^e^ | 25.63±0.10 ^bc^ | 2.49±0.08 ^bc^ | 5.68±0.17 ^bc^ | 22.76±0.80 ^c^ |
| R5 | 1.59±0.05 ^ghi^ | 0.27±0.005 ^bcd^ | 10.82±0.08 ^efg^ | 27.24±1.56 ^bc^ | 25.52±1.52 ^bc^ | 2.64±0.51 ^ab^ | 6.03 ±1.16^ab^ | 26.73±0.67 ^a^ |
| R6 | 1.75±0.08 ^cdef^ | 0.27±0.009 ^bcd^ | 11.20±0.03 ^bc^ | 24.37±0.42 ^ef^ | 25.25±0.21 ^cd^ | 3.13±0.17 ^a^ | 7.16±0.39 ^a^ | 26.30±0.38 ^a^ |
| R7 | 1.63±0.03 ^efgh^ | 0.27±0.008 ^de^ | 11.06±0.07 ^cde^ | 23.72±0.67 ^fg^ | 25.45±0.16 ^bc^ | 1.59±0.26 ^efg^ | 3.64±0.60 ^efg^ | 21.66±0.16 ^d^ |

mgGA/g= mg gallic acid equivalent/g coffee powder; mgCGA/g= mg chlorogenic acid equivalent/g coffee powder.

Data are expressed as mean ± standard deviation*.* Values in the same column followed by a different superscript letter (a to j) are statistically different (p<0.05), following pairwise comparison by Tukey’s HSD test.

**Table S3**. Complete headspace compositions of the whole (W) and ground (G) Arabica samples (A1 to A4).

| **Compound** | **l.r.i.^a^** | **Relative abundance (%) ± SD** | | | | | | | |
| --- | --- | --- | --- | --- | --- | --- | --- | --- | --- |
|  |  | **A1_W** | **A1_G** | **A2_W** | **A2_G** | **A3_W** | **A3_G** | **A4_W** | **A4_G** |
| formic acid | 537 | -^b^ | - | - | - | 0.2±0.33 | - | - | 0.4±0.21 |
| acetic acid | 603 | 2.9±0.18 | 4.1±0.02 | 2.0±0.35 | 3.7±0.4 | 2.3±0.03 | 3.7±1.39 | 2.1±0.28 | 5.9±1.12 |
| ethyl acetate | 616 | - | - | - | - | - | - | 0.2±0.35 | - |
| 1-hydroxy-2-propanone (syn. Acetol) | 656 | 0.4±0.04 | 0.7±0.06 | - | 0.6±0.01 | 0.2±0.21 | 0.6±0.13 | 0.2±0.24 | 0.8±0.15 |
| 3-methyl-2-butanol | 673 | - | - | - | 0.3±0.01 | 0.2±0.21 | - | - | 0.3±0.05 |
| 3-hydroxy-2-butanone (syn. Acetoin) | 707 | 0.2±0.28 | 0.2±0.21 | - | - | - | - | - | 0.2±0.21 |
| pyrazine | 737 | - | 0.3±0.03 | - | - | 0.2±0.21 | 0.2±0.34 | - | - |
| pyridine | 747 | 8.4±2.67 | 8.1±1.04 | 3.6±1.45 | 6±0.02 | 5.9±2.14 | 7.0±2.42 | 5.3±1.06 | 6.5±0.70 |
| 1-methylpiperidine | 777 | 0.7±0.30 | 0.5±0.13 | 1.0±0.33 | 0.4±0.00 | 1.0±0.01 | 0.7±0.49 | 1.1±0.01 | 0.6±0.12 |
| 3,5-dimethylisoxazole | 801 | - | - | - | - | - | - | - | - |
| 3-hexanol | 806 | - | - | - | - | 0.2±0.31 | - | - | - |
| dihydro-2-methyl-3(2H)-furanone | 811 | 1.7±0.52 | 1.1±0.15 | - | 0.6±0.07 | 0.5±0.71 | 0.6±0.33 | 0.8±0.11 | 0.8±0.11 |
| 2-methylpyridine | 819 | - | - | - | - | 0.2±0.28 | - | - | - |
| 2-methylpyrazine | 830 | 4.5±1.18 | 5.3±0.28 | 3.2±1.06 | 4.8±0.25 | 4.5±1.10 | 5.9±0.52 | 3.3±0.74 | 3.6±0.42 |
| 3-methylbutyric acid | 832 | - | - | - | - | - | - | - | - |
| furfural | 834 | 2.1±0.08 | 2.4±0.14 | 1.1±0.77 | 1.7±0.43 | 1.5±0.15 | 2.0±0.03 | 2.1±0.61 | 2.6±0.18 |
| ethyl-2-methyl butyrate | 847 | - | - | - | - | - | - | - | 0.2±0.32 |
| furfuryl alcohol (syn. 2-furanmethanol) | 858 | 11.2±1.38 | 9.8±0.59 | 11.6±0.96 | 11.5±0.53 | 11.1±0.1 | 11.4±4.41 | 13.7±0.71 | 14±0.76 |
| 3-methylpyridine | 866 | - | - | - | - | - | 0.2±0.23 | - | - |
| acetoxyacetone (syn. 1-(acetyloxy)-2-propanone | 867 | 3.9±0.42 | 3.2±0.02 | 2.1±0.83 | 2.6±0.18 | 2.9±0.13 | 2.9±0.50 | 3.7±0.35 | 3.5±0.13 |
| furfuryl formate | 904 | 0.8±0.14 | 0.6±0.03 | - | 0.4±0.03 | 0.6±0.06 | 0.5±0.13 | 0.5±0.01 | 0.7±0.16 |
| (*E,E*)-2,4-hexadienal | 911 | - | - | - | - | - | - | - | - |
| 2,6-dimethylpyrazine | 913 | 9.7±2.57 | 1.0±.084 | 8.1±1.36 | 9.8±0.24 | 9.6±1.00 | 8.4±3.24 | 8.7±1.42 | 7.7±0.18 |
| 2-acetyl furan | 914 | - | - | - | - | - | 3.3±4.60 | - | - |
| γ-butyrolactone | 915 | 1.1±1.57 | 1.0±0.28 | 1.6±0.24 | 1.4±0.49 | 1.5±0.14 | 1.6±2.19 | 2.2±0.37 | 1.8±0.78 |
| 2-ethylpyrazine | 916 | 1.6±0.86 | 2.6±0.01 | 1.9±0.21 | 2.9±0.23 | 2.5±0.09 | 3.5±0.80 | 2.0±0.03 | 2.6±1.75 |
| 2,3-dimethylpyrazine | 923 | 1.8±1.31 | 1.4±0.21 | 0.9±0.08 | 1.3±0.03 | 1.0±0.13 | 1.6±0.12 | 0.9±0.28 | 1.0±0.12 |
| 2,5-hexanedione (syn. Acetonyl acetone) | 926 | 0.2±0.32 | 0.5±0.02 | - | - | - | 0.3±0.35 | 0.2±0.33 | 0.5±0.13 |
| 2,4-dimethylpyridine | 927 | - | 0.2±0.21 | - | - | - | - | - | - |
| 3-methyl-2,5-furandione | 944 | - | - | - | - | - | - | - | 0.5±0.19 |
| 4-ethylpyridine | 956 | 0.8±0.04 | 0.7±0.06 | 0.9±0.13 | 0.7±0.00 | 0.9±0.04 | 0.9±0.11 | 1.0±0.05 | 0.8±0.04 |
| 1-acetoxy-2-butanone | 960 | 1.3±0.11 | 0.9±0.00 | 0.8±0.16 | 0.7±0.01 | 0.9±0.04 | 0.8±0.14 | 1.1±0.03 | 1.0±0.10 |
| 5-methylfurfural | 964 | 5.2±0.70 | 3.9±0.19 | 3.6±0.12 | 3.3±0.05 | 3.6±0.16 | 3.5±1.19 | 4.3±0.15 | 4.5±0.06 |
| phenol | 983 | 0.3±0.35 | 0.2±0.29 | 0.3±0.36 | 0.2±0.25 | 0.2±0.27 | - | - | 0.2±0.23 |
| 2-pentyl furan | 992 | - | - | - | - | 0.2±0.23 | - | - | 0.2±0.23 |
| 2-furfuryl acetate | 995 | 8.8±1.14 | 6.6±0.33 | 6.7±0.11 | 5.3±0.33 | 8.0±0.28 | 6.2±0.31 | 9.1±0.78 | 8.1±0.56 |
| 2-ethyl-6-methylpyrazine | 999 | 2.7±0.12 | 3.5±0.11 | 4.0±0.00 | 4.2±0.06 | 3.6±0.18 | 2.2±3.15 | 2.6±0.08 | 2.5±0.08 |
| 2-ethyl-5-methylpyrazine | 1002 | 1.5±0.14 | 2.0±0.01 | 2.5±0.13 | 2.4±0.02 | 1.8±0.17 | 2.5±0.64 | 1.5±0.01 | 1.5±0.06 |
| trimethylpyrazine | 1003 | 2.0±0.47 | 2.4±0.30 | 3±0.06 | 2.9±0.00 | 2.9±0.51 | 2.6±0.04 | 2.1±0.23 | 1.8±0.21 |
| 2-ethyl-3-methylpyrazine | 1005 | 0.6±0.44 | 1.2±0.10 | 1.5±0.13 | 1.6±0.12 | 1.3±0.30 | 1.5±0.53 | 0.7±0.30 | 0.7±0.16 |
| 3-methoxypyridine | 1006 | 0.8±1.20 | 1.3±0.27 | 1.8±0.00 | - | 0.5±0.64 | - | 1.4±0.40 | 1.3±0.33 |
| 1-(2-furanyl)-1-propanone (syn 2-furylethyl ketone) | 1008 | 0.2±0.29 | 0.2±0.25 | - | - | 0.2±0.24 | 0.2±0.28 | 0.2±0.30 | 0.2±0.25 |
| 2-propylpyrazine | 1009 | - | - | - | - | - | 0.7±1.00 | - | - |
| 2-vinyl-6-methylpyrazine | 1015 | - | 0.4±0.06 | - | 0.2±0.21 | 0.2±0.23 | 0.2±0.28 | 0.2±0.28 | - |
| 2-acetylpyrazine | 1022 | - | 0.6±0.13 | 0.5±0.64 | 0.7±0.09 | 0.6±0.17 | 0.4±0.52 | - | - |
| 5-ethyl-2-methylpyridine | 1023 | - | - | - | - | - | - | - | - |
| 3-hydroxy-2-methyl-2-cyclopenten-1-one | 1024 | 0.4±0.54 | 0.5±0.02 | 0.8±0.17 | 0.4±0.01 | 0.6±0.02 | 0.2±0.27 | 0.8±0.04 | 0.6±0.07 |
| limonene | 1032 | - | - | - | - | - | - | - | 0.2±0.21 |
| 2,3-dimethyl-2-cyclopentenone | 1038 | 0.6±0.78 | 0.6±0.25 | 0.8±0.25 | 0.6±0.10 | 0.8±0.23 | 0.3±0.46 | 0.5±0.74 | 0.8±0.24 |
| 2-acetylpyrrole | 1062 | 1.5±0.07 | 1.0±0.03 | 0.9±1.30 | 1.2±0.06 | 1.5±0.05 | 1.0±0.02 | 1.5±0.01 | 1.3±0.06 |
| 2,3,4-trimethyl-2-cyclopenten-1-one | 1063 | - | 0.4±0.05 | 0.6±0.07 | 0.2±0.29 | 0.4±0.03 | 0.2±0.23 | 0.3±0.35 | 0.5±0.03 |
| *cis*-linalool oxide (furanoid) | 1073 | 0.4±0.57 | 0.3±0.35 | 0.3±0.35 | 0.2±0.28 | 0.3±0.42 | 0.3±0.35 | 0.4±0.57 | 1.1±0.46 |
| 2-amino-6-methylpyridine (syn. 6-methyl-2-pyridinamine) | 1074 | 1.3±0.47 | 1.0±0.40 | 1.4±0.24 | 0.6±0.19 | 1.1±0.63 | 0.7±0.23 | 1.3±0.61 | 0.3±0.42 |
| 2,6-diethylpyrazine | 1083 | 2.1±0.12 | 2.9±0.32 | 4.6±0.48 | 3.9±0.55 | 3.4±0.10 | 3.5±0.41 | 2.2±0.34 | 2.0±0.42 |
| 2-ethyl-3,5-dimethyl pyrazine | 1084 | - | - | 1.3±0.28 | 1.0±0.01 | - | - | - | - |
| 2-furfurylfuran | 1085 | 1.1±0.12 | 1.2±0.11 | - | - | 1.3±0.07 | 1.1±0.16 | 1.2±0.08 | 1.1±0.18 |
| 2,3-diethylpyrazine | 1086 | 0.2±0.32 | 0.8±0.05 | 1.3±0.14 | 0.6±0.78 | 0.7±0.05 | 1.0±0.13 | 0.2±0.34 | 0.4±0.09 |
| *o*-guaiacol | 1090 | 1.3±0.24 | 1.2±0.08 | 1.0±0.13 | 1.4±0.06 | 1.0±0.02 | 1.0±0.18 | 1.1±0.18 | 1.2±0.28 |
| 3-ethyl-2-hydroxy-2-cyclopenten-1-one | 1091 | 0.9±0.18 | 0.7±0.06 | 0.8±0.14 | 0.5±0.08 | 0.7±0.11 | 0.6±0.06 | 1.0±0.20 | 0.7±0.22 |
| 2-methyl-3-propylpyrazine | 1092 | - | - | - | - | - | - | - | - |
| 4,4-dimethyl-2-cyclohexen-1-one | 1102 | - | 0.3±0.35 | - | - | - | - | - | - |
| nonanal | 1104 | - | - | - | - | - | - | - | - |
| *iso*propenylpyrazine | 1107 | 1.0±0.15 | 1.0±0.23 | 1.6±0.18 | 0.9±0.05 | 1.2±0.09 | 0.9±0.23 | 1.0±0.01 | 0.8±0.11 |
| maltol | 1111 | 2.5±0.27 | 2.1±0.06 | 3.3±1.05 | 1.5±0.30 | 3.0±0.83 | 1.3±0.00 | 3.2±1.78 | 2.4±0.09 |
| phenylethyl alcohol | 1112 | - | - | - | - | - | - | - | - |
| 4-methyl-2-aminopyridine | 1113 | 0.2±0.30 | - | - | - | - | - | - | - |
| 3,4,4-trimethyl-2-cyclopenten-1-one | 1129 | 0.2±0.30 | - | - | - | 0.2±0.23 | - | - | 0.2±0.35 |
| methyl nicotinate | 1138 | - | 0.2±0.23 | 0.7±0.27 | 0.2±0.28 | 0.4±0.13 | 0.4±0.13 | 0.2±0.34 | 0.3±0.35 |
| 5H-5-methyl-6,7-dihydrocyclopentapyrazine | 1141 | - | 0.2±0.32 | 0.9±0.31 | 0.6±0.02 | 0.5±0.04 | 0.6±0.01 | - | 0.2±0.28 |
| 2,3-diethyl-5-methylpyrazine | 1157 | - | - | - | 0.2±0.25 | - | - | - | - |
| 3,5-diethyl-2-methylpyrazine | 1158 | 0.4±0.59 | 0.7±0.20 | 1.4±0.34 | 1.0±0.03 | 0.8±0.28 | 0.7±0.25 | 0.5±0.13 | 0.4±0.58 |
| 2,3,5-trimethyl-6-ethylpyrazine | 1165 | - | - | - | - | - | 0.2±0.22 | - | - |
| 2-furfuryl-5-methylfuran | 1184 | 0.6±0.13 | 0.6±0.06 | 0.4±0.57 | 0.6±0.34 | 0.8±0.10 | 0.5±0.21 | 0.8±0.22 | 0.6±0.13 |
| 1-furfurylpyrrole | 1185 | 1.0±0.01 | 0.8±0.08 | 1.7±0.01 | 0.5±0.64 | 0.9±0.04 | 0.8±0.03 | 1.1±0.12 | 0.8±0.00 |
| 2-butanoyl-5-methylfuran | 1188 | - | 0.7±0.06 | - | 0.3±0.35 | - | 0.2±0.33 | 0.7±0.11 | 0.4±0.50 |
| 2-methyl-5[(*Z*)-1-propenyl]pyrazine | 1192 | 0.4±0.52 | 0.3±0.41 | 0.8±0.20 | 0.6±0.08 | - | 0.6±0.35 | - | 0.2±0.30 |
| methyl salicylate | 1193 | - | - | - | 0.2±0.24 | - | - | - | 0.2±0.28 |
| verbenone | 1205 | - | - | 0.3±0.36 | 0.2±0.23 | - | 0.2±0.23 | 0.2±0.29 | - |
| 2,4-dimethylacetophenone | 1220 | - | - | - | - | - | - | - | - |
| 2,5-dimethyl-6,7-dihydro-(5H)-cyclopentapyrazine | 1222 | - | - | 0.4±0.54 | - | 0.3±0.47 | - | - | - |
| furfuryl 3-methylbutanoate | 1223 | 0.3±0.42 | 0.3±0.37 | - | 0.3±0.42 | - | 0.3±0.39 | 0.6±0.01 | 0.3±0.40 |
| 2-methyl-6-*iso*pentylpyrazine | 1249 | - | - | - | - | - | - | - | - |
| 2-phenyl-2-butenal | 1279 | - | - | - | 0.2±0.24 | - | 0.2±0.21 | - | - |
| *p*-ethylguaiacol | 1280 | 0.2±0.30 | 0.9±0.08 | 1.2±0.01 | 1.3±0.10 | 0.9±0.07 | 1.1±0.24 | 0.9±0.15 | 0.6±0.02 |
| (E)-anethole | 1283 | - | 0.2±0.26 | - | 0.2±0.27 | - | 0.2±0.25 | - | 0.2±0.35 |
| difurfuryl ether | 1302 | 0.5±0.77 | 0.6±0.13 | 1.1±0.13 | 0.5±0.1 | 0.8±0.03 | 0.6±0.18 | 0.9±0.12 | 0.8±0.25 |
| *p*-vinylguaiacol | 1314 | 1.1±1.51 | 2.4±0.21 | 3.5±0.61 | 4.7±0.58 | 2±0.13 | 2.2±0.32 | 2.4±0.33 | 1.8±0.14 |
| 4-vinylveratrole | 1367 | - | - | - | 1±0.31 | - | - | - | - |
| €-β-damascenone | 1382 | - | 0.2±0.26 | - | 0.5±0.1 | - | 0.2±0.30 | - | - |
| β-bourbonene | 1385 | 0.2±0.30 | - | - | - | - | - | - | - |
|  |  |  |  |  |  |  |  |  |  |
| Acids/esters | | 2.9±0.18 | 4.1±0.02 | 2.0±0.35 | 3.7±0.40 | 2.5±0.30 | 3.7±1.39 | 2.3±0.06 | 6.6±0.59 |
| Terpenes | | 0.6±0.27 | 0.3±0.35 | 0.5±0.01 | 0.4±0.05 | 0.3±0.42 | 0.4±0.13 | 0.6±0.28 | 1.3±0.25 |
| Aldehydes-ketones | | 8.1±0.98 | 7.8±0.22 | 5.8±0.86 | 5.8±0.72 | 6.6±0.01 | 6.0±0.03 | 7.7±1.10 | 8.8±0.19 |
| Pyrazines | | 28.5±0.71 | 35.5±0.72 | 37.8±3.09 | 39.5±0.68 | 35.0±0.40 | 37±12.52 | 25.8±0.23 | 25.3±0.71 |
| Pyridines-piperidines | | 12.3±0.37 | 11.9±0.17 | 9.4±1.88 | 8.0±0.07 | 10±2.24 | 9.8±3.13 | 10.3±2.34 | 9.7±0.36 |
| Pyrroles | | 2.4±0.06 | 1.8±0.11 | 2.6±1.32 | 1.7±0.58 | 2.4±0.01 | 1.8±0.05 | 2.6±0.13 | 2.1±0.06 |
| Phenols | | 2.9±1.92 | 4.6±0.25 | 5.9±0.39 | 7.8±1.22 | 4.1±0.23 | 4.3±0.38 | 4.4±0.01 | 4.0±0.40 |
| Alcohols | | - | - | - | 0.3±0.01 | 0.4±0.10 | - | - | 0.3±0.05 |
| Furans | | 33.7±0.72 | 28.7±0.68 | 26.2±0.64 | 25.9±0.47 | 29.9±0.93 | 31.8±11.73 | 37.3±0.78 | 36.3±0.69 |
| Apocarotenes | | - | 0.2±0.26 | - | 0.5±0.10 | - | 0.2±0.30 | - | - |
| Others | | 2.5±0.27 | 2.2±0.20 | 3.3±1.05 | 2.7±0.28 | 3±0.83 | 1.5±0.25 | 3.2±1.78 | 2.6±0.44 |
|  |  |  |  |  |  |  |  |  |  |
| Total identified (%) | | 93.9±2.40 | 97.1±1.53 | 93.4±4.73 | 96.1±1.44 | 94.2±2.53 | 96.5±3.35 | 94.2±0.30 | 96.9±0.10 |
| ^a^ Linear retention indices on a HP5-MS capillary column; ^b^ Not detected. Legend: W, whole; G, ground. | | | | | | | | | |

**Table S4**. Complete headspace compositions of the whole (W) and ground (G) Arabica samples (A5 to A9).

| **Compound** | **l.r.i.^a^** | **Relative abundance (%) ± SD** | | | | | | | | | |
| --- | --- | --- | --- | --- | --- | --- | --- | --- | --- | --- | --- |
|  |  | **A5_W** | **A5_G** | **A6_W** | **A6_G** | **A7_W** | **A7_G** | **A8_W** | **A8_G** | **A9_W** | **A9_G** |
| formic acid | 537 | -^b^ | - | - | 0.2±0.21 | - | - | - | - | - | - |
| acetic acid | 603 | 2.6±0.07 | 4.2±1.66 | 2.4±0.11 | 4±2.11 | 1±0.25 | 3.4±0.34 | 2.0±0.01 | 4.5±0.95 | 2.6±0.26 | 6.2±1.43 |
| ethyl acetate | 616 | - | - | - | - | - | - | - | - | - | - |
| 1-hydroxy-2-propanone (syn. Acetol) | 656 | 0.2±0.28 | 0.6±0.11 | 0.1±0.14 | 0.5±0.06 | 0.1±0.14 | 0.5±0.01 | - | 0.6±0.04 | 0.1±0.20 | 0.5±0.00 |
| 3-methyl-2-butanol | 673 | - | 0.2±0.21 | - | - | - | - | - | - | - | - |
| 3-hydroxy-2-butanone (syn. Acetoin) | 707 | - | 0.2±0.23 | - | - | - | - | - | 0.2±0.21 | - | - |
| pyrazine | 737 | 0.2±0.21 | 0.3±0.37 | - | 0.5±0.08 | 0.1±0.14 | - | - | 0.4±0.02 | - | - |
| pyridine | 747 | 5.6±2.47 | 8.0±3.22 | 3.4±0.23 | 7.3±0.74 | 4.2±0.31 | 4.3±0.23 | 3.3±1.05 | 8.0±1.69 | 6.3±0.49 | 4.5±1.41 |
| 1-methylpiperidine | 777 | 0.9±0.00 | 0.8±0.46 | 0.5±0.19 | 0.3±0.42 | 0.4±0.04 | 0.2±0.29 | 0.8±0.04 | 0.6±0.23 | 0.7±0.12 | 0.4±0.00 |
| 3,5-dimethylisoxazole | 801 | - | 0.6±0.89 | - | 0.4±0.49 | - | 0.2±0.34 | - | - | - | 0.3±0.37 |
| 3-hexanol | 806 | - | - | - | - | - | - | - | - | 0.1±0.11 | - |
| dihydro-2-methyl-3(2H)-furanone | 811 | 0.8±0.42 | 0.8±0.62 | 0.6±0.04 | 1.0±0.23 | 0.3±0.00 | - | 0.4±0.11 | 0.8±0.12 | 0.9±0.09 | 0.4±0.14 |
| 2-methylpyridine | 819 | - | 0.2±0.23 | - | 0.2±0.21 | 0.4±0.15 | - | 0.2±0.21 | - | 0.3±0.05 | - |
| 2-methylpyrazine | 830 | 4.6±1.20 | 4.7±1.03 | 4.2±0.12 | 6.3±0.01 | 5.4±0.69 | 4.7±0.35 | 4.3±0.91 | 5.8±0.60 | 4.5±0.44 | 3.2±0.86 |
| 3-methylbutyric acid | 832 | - | - | 0.3±0.35 | - | - | - | 0.3±0.35 | - | - | - |
| furfural | 834 | 1.9±0.35 | 1.9±0.38 | 1.8±0.18 | 1.6±0.05 | 0.8±0.10 | 1.3±0.18 | 1.3±0.21 | 1.9±0.24 | 1.8±0.04 | 1.7±0.21 |
| ethyl-2-methyl butyrate | 847 | - | - | - | - | - | - | - | - | - | - |
| furfuryl alcohol (syn. 2-furanmethanol) | 858 | 12.2±1.2 | 11.0±3.13 | 12.4±1.65 | 7.9±2.48 | 8.2±1.85 | 10.6±1.32 | 10.6±0.31 | 10.3±0.54 | 10.2±1.12 | 13.2±0.29 |
| 3-methylpyridine | 866 | - | - | - | - | 0.1±0.14 | - | - | - | 0.1±0.18 | - |
| acetoxyacetone (syn. 1-(acetyloxy)-2-propanone | 867 | 2.9±0.21 | 2.8±0.11 | 3±0.05 | 2.4±0.28 | 2.1±0.24 | 1.9±0.2 | 2.2±0.04 | 2.6±0.25 | 3.1±0.09 | 3.5±0.52 |
| furfuryl formate | 904 | 0.5±0.14 | 0.5±0.04 | 0.6±0.06 | 0.4±0.11 | 0.4±0.06 | 0.3±0.04 | 0.4±0.01 | 0.4±0.02 | 0.4±0.04 | 0.4±0.11 |
| (*E,E*)-2,4-hexadienal | 911 | - | - | - | - | - | - | - | - | - | - |
| 2,6-dimethylpyrazine | 913 | 9.8±1.84 | 9.3±0.37 | 10.6±0.01 | 11.6±0.4 | 11.0±0.30 | 9.5±0.67 | 11±0.35 | 9.9±0.56 | 10.7±1.12 | 9.8±0.52 |
| 2-acetyl furan | 914 | - | - | - | - | - | - | - | - | - | - |
| γ-butyrolactone | 915 | 1.9±0.14 | 1.7±0.8 | 0.9±1.20 | 0.8±0.33 | 0.5±0.71 | 1.8±0.49 | 0.6±0.78 | 1.4±0.06 | 1.6±0.18 | 2.6±0.02 |
| 2-ethylpyrazine | 916 | 2.6±0.35 | 2.5±0.37 | 2.5±0.11 | 3.0±0.04 | 3.3±0.28 | 2.6±0.19 | 2.8±0.39 | 2.8±0.65 | 2.4±0.36 | 2.2±0.11 |
| 2,3-dimethylpyrazine | 923 | 1.0±0.28 | 1.2±0.18 | 1.1±0.35 | 1.7±0.60 | 1.7±0.06 | 1.4±0.01 | 1.3±0.10 | 1.3±0.17 | 1.1±0.11 | 1.2±0.18 |
| 2,5-hexanedione (syn. Acetonyl acetone) | 926 | - | 0.4±0.03 | 0.3±0.04 | 0.3±0.35 | - | - | - | 0.2±0.28 | - | 0.2±0.29 |
| 2,4-dimethylpyridine | 927 | - | - | - | 0.2±0.23 | - | - | - | - | - | - |
| 3-methyl-2,5-furandione | 944 | - | 0.2±0.21 | 0.2±0.21 | - | - | - | - | - | - | 0.2±0.25 |
| 4-ethylpyridine | 956 | 0.8±0.07 | 0.9±0.02 | 0.7±0.03 | 0.6±0.06 | 0.8±0.03 | 0.6±0.04 | 0.7±0.06 | 0.7±0.04 | 0.9±0.00 | 0.9±0.06 |
| 1-acetoxy-2-butanone | 960 | 0.9±0.07 | 0.8±0.00 | 1.0±0.01 | 0.7±0.04 | 0.5±0.07 | 0.5±0.07 | 0.9±0.08 | 0.7±0.04 | 1.1±0.04 | 1.0±0.20 |
| 5-methylfurfural | 964 | 4.0±0.07 | 3.3±0.54 | 4.5±0.47 | 3.0±0.45 | 2.0±0.3 | 2.1±0.11 | 3.4±0.34 | 3.3±0.16 | 3.9±0.13 | 4.5±0.57 |
| phenol | 983 | - | - | - | - | 0.3±0.36 | 0.4±0.00 | 0.2±0.31 | - | 0.3±0.39 | - |
| 2-pentyl furan | 992 | - | 0.2±0.24 | - | 0.2±0.28 | - | - | - | - | 0.2±0.28 | - |
| 2-furfuryl acetate | 995 | 7.4±0.28 | 7.3±0.64 | 7.6±0.65 | 5.5±0.27 | 4.5±0.13 | 4.6±0.01 | 6.3±0.15 | 6.5±0.6 | 8.3±0.22 | 7.4±1.39 |
| 2-ethyl-6-methylpyrazine | 999 | 3.8±0.14 | 3.6±0.16 | 3.8±0.01 | 4.7±0.25 | 5±0.54 | 4.9±0.01 | 4.5±0.16 | 4.7±0.35 | 3.6±0.09 | 3.4±0.7 |
| 2-ethyl-5-methylpyrazine | 1002 | 2.0±0.21 | 2.1±0.05 | 1.9±0.18 | 3.1±0.38 | 2.7±0.59 | 3.0±0.12 | 2.4±0.12 | 2.4±0.06 | 1.9±0.18 | 2.0±0.28 |
| trimethylpyrazine | 1003 | 3.1±0.00 | 2.5±0.37 | 2.8±0.30 | 3.1±0.28 | 3.7±0.64 | 3.8±0.14 | 3.4±0.13 | 3.5±0.58 | 2.8±0.43 | 2.5±0.54 |
| 2-ethyl-3-methylpyrazine | 1005 | 1.1±0.14 | 1.3±0.04 | 1.2±0.43 | 2.1±0.05 | 2.0±0.24 | 1.9±0.16 | 1.7±0.09 | 1.8±0.31 | 1.1±0.28 | 1.3±0.19 |
| 3-methoxypyridine | 1006 | 1.3±0.49 | 0.7±0.93 | 1.2±0.20 | 0.7±0.41 | - | - | 1.5±0.40 | 0.3±0.42 | 1.3±0.28 | 1.1±0.66 |
| 1-(2-furanyl)-1-propanone (syn 2-furylethyl ketone) | 1008 | 0.2±0.21 | 0.2±0.23 | 0.2±0.28 | 0.2±0.24 | - | - | 0.2±0.24 | 0.2±0.34 | 0.2±0.25 | 0.3±0.00 |
| 2-propylpyrazine | 1009 | - | - | - | - | - | 0.7±0.98 | - | 1.0±1.35 | - | - |
| 2-vinyl-6-methylpyrazine | 1015 | 0.2±0.21 | 0.3±0.05 | 0.4±0.03 | 0.4±0.11 | 0.4±0.04 | 0.4±0.03 | 0.3±0.04 | 0.5±0.11 | 0.4±0.06 | 0.3±0.00 |
| 2-acetylpyrazine | 1022 | 0.3±0.42 | - | 0.3±0.39 | 0.5±0.01 | - | 0.6±0.37 | 0.4±0.62 | 0.5±0.66 | 0.3±0.42 | 0.7±0.09 |
| 5-ethyl-2-methylpyridine | 1023 | - | - | - | 0.2±0.23 | - | - | - | - | - | - |
| 3-hydroxy-2-methyl-2-cyclopenten-1-one | 1024 | 0.8±0.07 | 0.4±0.02 | 0.7±0.02 | 0.4±0.04 | 0.5±0.04 | 0.4±0.03 | 0.5±0.06 | 0.4±0.17 | 0.6±0.03 | 0.5±0.05 |
| limonene | 1032 | - | - | - | - | - | - | - | 0.2±0.23 | - | - |
| 2,3-dimethyl-2-cyclopentenone | 1038 | 0.7±0.28 | 0.7±0.01 | 0.5±0.64 | 0.7±0.02 | - | 0.5±0.07 | 0.6±0.23 | 0.6±0.26 | 0.5±0.16 | 0.9±0.06 |
| 2-acetylpyrrole | 1062 | 1.7±0.28 | 1.2±0.17 | 1.7±0.16 | 1.1±0.18 | 1.5±0.03 | 1.3±0.16 | 1.7±0.08 | 1.1±0.08 | 1.4±0.05 | 1.3±0.16 |
| 2,3,4-trimethyl-2-cyclopenten-1-one | 1063 | - | 0.2±0.30 | - | 0.4±0.04 | - | 0.4±0.01 | 0.4±0.06 | 0.2±0.28 | 0.4±0.06 | 0.3±0.38 |
| *cis*-linalool oxide (furanoid) | 1073 | 0.2±0.28 | 0.5±0.17 | 0.3±0.35 | 0.5±0.04 | - | - | 0.3±0.35 | 0.3±0.35 | 0.3±0.35 | 0.6±0.14 |
| 2-amino-6-methylpyridine (syn. 6-methyl-2-pyridinamine) | 1074 | 1.2±0.49 | 0.8±0.04 | 1.2±0.38 | 0.5±0.00 | 0.9±0.11 | 0.5±0.13 | 1.3±0.45 | 1±0.29 | 1.3±0.29 | 0.9±0.04 |
| 2,6-diethylpyrazine | 1083 | 3.8±0.07 | 3.5±0.60 | 4.1±0.18 | 4.7±0.24 | 5.5±0.76 | 5.6±0.85 | 4.8±0.51 | 4.1±0.5 | 3.1±0.43 | 3.1±0.25 |
| 2-ethyl-3,5-dimethyl pyrazine | 1084 | 1.4±0.07 | - | - | - | 1.5±0.28 | - | 1.2±0.08 | 1.0±0.08 | - | - |
| 2-furfurylfuran | 1085 | 0.4±0.49 | 1.2±0.13 | 1.4±0.28 | 1.4±0.07 | - | 1.5±0.13 | - | - | 1.3±0.17 | 1.2±0.24 |
| 2,3-diethylpyrazine | 1086 | 0.4±0.57 | 0.9±0.11 | 0.3±0.49 | 1.3±0.15 | 1.7±0.36 | 1.7±0.01 | 1.2±0.25 | 0.9±0.16 | 0.3±0.45 | 0.5±0.71 |
| *o*-guaiacol | 1090 | 0.8±0.14 | 0.9±0.24 | 0.9±0.03 | 1.0±0.16 | 1.6±0.18 | 1.5±0.03 | 1.1±0.04 | 0.7±0.02 | 1.1±0.19 | 1.1±0.21 |
| 3-ethyl-2-hydroxy-2-cyclopenten-1-one | 1091 | 0.7±0.07 | 0.6±0.06 | 0.8±0.09 | 0.6±0.04 | 0.6±0.21 | - | 0.7±0.01 | 0.3±0.42 | 0.8±0.07 | 0.3±0.38 |
| 2-methyl-3-propylpyrazine | 1092 | - | - | - | - | - | 0.5±0.05 | - | 0.2±0.30 | - | - |
| 4,4-dimethyl-2-cyclohexen-1-one | 1102 | - | - | - | - | - | - | - | - | - | - |
| nonanal | 1104 | - | - | - | - | - | - | - | - | 0.2±0.27 | - |
| *iso*propenylpyrazine | 1107 | 1.1±0.07 | 1.0±0.06 | 1.1±0.01 | 1.0±0.07 | 1.1±0.02 | 1.1±0.06 | 1.0±0.02 | 0.9±0.03 | 1.0±0.06 | 1.0±0.04 |
| maltol | 1111 | 2.2±0.21 | 2.0±0.71 | 2.7±0.15 | 1.2±0.08 | 1.0±0.08 | 1.5±0.03 | 1.6±0.35 | 1.5±0.05 | 2.7±0.08 | 1.9±0.34 |
| phenylethyl alcohol | 1112 | - | - | - | - | - | - | 0.4±0.57 | - | - | - |
| 4-methyl-2-aminopyridine | 1113 | - | - | - | - | - | - | - | - | - | - |
| 3,4,4-trimethyl-2-cyclopenten-1-one | 1129 | - | - | - | - | - | - | 0.2±0.34 | - | - | - |
| methyl nicotinate | 1138 | 0.3±0.35 | 0.4±0.08 | 0.2±0.25 | - | 0.2±0.25 | 0.4±0.04 | 0.4±0.05 | - | 0.2±0.27 | 0.2±0.23 |
| 5H-5-methyl-6,7-dihydrocyclopentapyrazine | 1141 | 0.4±0.49 | 0.6±0.16 | 0.3±0.41 | 0.5±0.05 | 0.7±0.01 | 0.8±0.04 | 0.7±0.05 | 0.5±0.09 | 0.2±0.33 | 0.2±0.3 |
| 2,3-diethyl-5-methylpyrazine | 1157 | - | - | - | - | 0.4±0.13 | 0.2±0.35 | 0.2±0.21 | - | - | - |
| 3,5-diethyl-2-methylpyrazine | 1158 | 0.7±0.14 | 0.8±0.26 | 0.7±0.00 | 0.8±0.2 | 1.3±0.2 | 1.1±0.25 | 1.1±0.31 | 0.8±0.14 | 0.8±0.29 | 0.7±0.28 |
| 2,3,5-trimethyl-6-ethylpyrazine | 1165 | - | - | - | 0.2±0.25 | 0.3±0.4 | 0.3±0.37 | - | - | - | - |
| 2-furfuryl-5-methylfuran | 1184 | 0.6±0.14 | 0.3±0.42 | 0.9±0.08 | 0.5±0.01 | 0.4±0.11 | 0.3±0.35 | 0.7±0.09 | 0.3±0.35 | 0.6±0.22 | 0.6±0.13 |
| 1-furfurylpyrrole | 1185 | 1.0±0.00 | 1.2±0.21 | 1.1±0.01 | 0.9±0.07 | 0.9±0.07 | 0.9±0.11 | 1.0±0.17 | 0.7±0.18 | 1.1±0.31 | 1.0±0.28 |
| 2-butanoyl-5-methylfuran | 1188 | 0.3±0.42 | 0.3±0.37 | 0.6±0.04 | - | 0.5±0.64 | - | 0.6±0.16 | - | - | 0.4±0.06 |
| 2-methyl-5[(*Z*)-1-propenyl]pyrazine | 1192 | 0.7±0.35 | 0.4±0.56 | 0.5±0.13 | 0.5±0.07 | 0.4±0.52 | 0.7±0.13 | 0.7±0.02 | 0.5±0.06 | 0.6±0.25 | 0.2±0.28 |
| methyl salicylate | 1193 | - | - | - | - | - | - | - | - | - | - |
| verbenone | 1205 | 0.3±0.35 | - | - | - | 0.3±0.40 | 0.2±0.28 | - | 0.2±0.23 | 0.2±0.26 | - |
| 2,4-dimethylacetophenone | 1220 | - | - | - | - | 0.3±0.35 | - | - | - | - | - |
| 2,5-dimethyl-6,7-dihydro-(5H)-cyclopentapyrazine | 1222 | 0.4±0.57 | - | - | - | 0.6±0.35 | - | - | 0.2±0.35 | - | - |
| furfuryl 3-methylbutanoate | 1223 | - | 0.3±0.35 | - | 0.3±0.36 | - | 0.4±0.61 | 0.3±0.35 | - | 0.2±0.35 | 0.3±0.48 |
| 2-methyl-6-*iso*pentylpyrazine | 1249 | - | - | - | - | 0.2±0.23 | 0.2±0.27 | - | - | - | - |
| 2-phenyl-2-butenal | 1279 | - | - | - | - | - | - | - | - | - | - |
| *p*-ethylguaiacol | 1280 | 0.8±0.28 | 0.7±0.04 | 0.5±0.67 | 0.9±0.03 | 2.9±0.14 | 2.3±0.11 | 1.1±0.23 | 0.6±0.05 | 0.7±0.08 | 0.8±0.01 |
| (E)-anethole | 1283 | 0.3±0.35 | 0.2±0.23 | - | 0.2±0.26 | - | 0.2±0.28 | - | - | - | - |
| difurfuryl ether | 1302 | 1.0±0.28 | 0.8±0.08 | 1.2±0.22 | 0.5±0.02 | 1.0±0.11 | 0.6±0 | 0.9±0.16 | 0.6±0.2 | 0.8±0.13 | 0.8±0.06 |
| *p*-vinylguaiacol | 1314 | 2.5±0.21 | 1.9±0.05 | 2.7±0.07 | 2.7±0.15 | 6±0.08 | 5.5±0.32 | 2.5±0.01 | 2.0±0.08 | 1.9±0.40 | 1.9±0.11 |
| 4-vinylveratrole | 1367 | - | - | - | - | - | 0.2±0.31 | - | - | - | - |
| (E)-β-damascenone | 1382 | - | 0.2±0.31 | - | 0.2±0.23 | - | 0.3±0.40 | - | - | - | - |
| β-bourbonene | 1385 | - | - | - | - | - | - | 0.2±0.28 | 0.2±0.33 | - | - |
|  |  |  |  |  |  |  |  |  |  |  |  |
| Acids/esters | | 2.6±0.07 | 4.2±1.66 | 2.7±0.47 | 4.2±2.32 | 1.0±0.25 | 3.4±0.34 | 2.2±0.36 | 4.5±0.95 | 2.6±0.26 | 6.2±1.43 |
| Terpenes | | 0.5±0.07 | 0.5±0.17 | 0.3±0.35 | 0.5±0.04 | 0.3±0.40 | 0.2±0.28 | 0.4±0.08 | 0.8±0.43 | 0.4±0.09 | 0.6±0.14 |
| Aldehydes-ketones | | 6.0±0.28 | 6.6±0.45 | 6.5±0.49 | 5.9±0.03 | 3.7±0.07 | 4.1±0.17 | 5.5±0.72 | 5.8±1.01 | 6.8±0.39 | 7.1±1.76 |
| Pyrazines | | 37.1±1.13 | 35.0±1.31 | 35.8±2.23 | 45.8±2.04 | 49.3±3.63 | 45.8±1.66 | 42.9±2.38 | 43.9±2.23 | 34.8±1.58 | 32.3±1.65 |
| Pyridines-piperidines | | 9.9±1.20 | 11.7±4.69 | 7.3±0.82 | 9.9±1.87 | 6.9±0.06 | 6.0±0.66 | 8.1±0.06 | 10.6±1.56 | 11.0±0.59 | 7.9±0.50 |
| Pyrroles | | 2.7±0.28 | 2.4±0.04 | 2.8±0.17 | 1.9±0.25 | 2.4±0.10 | 2.2±0.28 | 2.6±0.08 | 1.7±0.11 | 2.4±0.36 | 2.3±0.12 |
| Phenols | | 4.1±0.64 | 3.5±0.25 | 4.0±0.57 | 4.6±0.33 | 10.7±0.4 | 9.7±0.40 | 4.9±0.51 | 3.3±0.16 | 4.0±0.28 | 3.8±0.32 |
| Alcohols | | - | 0.2±0.21 | - | - | - | - | 0.4±0.57 | - | 0.1±0.11 | - |
| Furans | | 31±0.64 | 29.8±3.56 | 32.8±4.20 | 23.2±2.31 | 18.6±0.50 | 23.5±1.59 | 25.6±0.32 | 25.7±0.17 | 30.5±1.13 | 33.9±1.35 |
| Apocarotenes | | - | 0.2±0.31 | - | 0.2±0.23 | - | 0.3±0.40 | - | - | - | - |
| Others | | 2.4±0.57 | 2.8±0.41 | 2.7±0.15 | 1.7±0.83 | 1.3±0.44 | 2.1±0.91 | 1.6±0.35 | 1.5±0.05 | 2.7±0.08 | 2.2±0.71 |
|  |  |  |  |  |  |  |  |  |  |  |  |
| Total identified (%) | | 96.1±0.35 | 96.9±1.34 | 94.7±0.93 | 97.8±0.23 | 94.1±2.43 | 97.2±1.48 | 94.3±1.71 | 97.8±1.22 | 95.4±2.17 | 96.5±0.18 |
| ^a^ Linear retention indices on a HP5-MS capillary column; ^b^ Not detected. Legend: W, whole; G, ground. | | | | | | | | | | | |

**Table S5**. Complete headspace compositions of the whole (W) and ground (G) Robusta samples.

| **Compound** | **l.r.i.^a^** | **Relative abundance (%) ± SD** | | | | | | | | | | | | | |
| --- | --- | --- | --- | --- | --- | --- | --- | --- | --- | --- | --- | --- | --- | --- | --- |
|  |  | **R1_W** | **R1_G** | **R2_W** | **R2_G** | **R3_W** | **R3_G** | **R4_W** | **R4_G** | **R5_W** | **R5_G** | **R6_W** | **R6_G** | **R7_W** | **R7_G** |
| acetic acid | 603 | 1.1±0.18 | 4.6±0.07 | 1.2±0.13 | 3.3±0.14 | 1.2±0.15 | 3.5±1.35 | 1.7±0.11 | 3.7±0.05 | 1.1±0.28 | 2.6±0.58 | 1.3±0.37 | 2.4±0.2 | 0.9±0.11 | 2.8±0.03 |
| 1-hydroxy-2-propanone (syn. Acetol) | 656 | 0.1±0.14 | 0.6±0.02 | -^b^ | 0.4±0.06 | - | 0.4±0.04 | - | 0.7±0.08 | 0.1±0.14 | 0.5±0.15 | - | 0.4±0.01 | - | 0.4±0.04 |
| 3-methyl-2-butanol | 673 | - | 0.2±0.21 | - | - | 0.2±0.21 | - | 0.3±0.35 | - | - | - | - | - | - | - |
| pyrazine | 737 | 0.1±0.07 | - | - | 0.2±0.21 | - | 0.2±0.35 | - | 0.3±0.05 | 0.2±0.21 | 0.5±0.15 | - | 0.5±0.04 | - | 0.2±0.23 |
| pyridine | 747 | 4.0±0.88 | 5.1±0.60 | 2±1.04 | 7.7±0.92 | 2.2±0.66 | 4.3±2.55 | 2.5±0.66 | 7.3±0.13 | 3.5±0.21 | 7.4±2.02 | 2.3±0.06 | 6.3±0.9 | 3.5±1.78 | 4.4±0.81 |
| 1-methylpiperidine | 777 | 0.5±0.21 | 0.5±0.23 | 0.3±0.18 | 0.6±0.06 | - | 0.2±0.25 | 0.6±0.02 | 0.5±0.33 | 0.5±0.07 | 0.4±0.08 | 0.5±0.09 | 0.4±0.16 | 0.6±0.11 | 0.2±0.24 |
| 2,3-butanediol | 790 | - | - | 0.1±0.14 | - | - | - | 0.2±0.21 | - | - | - | - | - | - | - |
| 3,5-dimethylisoxazole | 801 | - | - | - | 0.5±0.74 | - | 0.3±0.37 | - | - | - | - | - | - | - | 0.3±0.42 |
| hexanal | 802 | - | - | - | - | - | - | - | - | 0.4±0.49 | - | - | - | - | - |
| dihydro-2-methyl-3(2H)-furanone | 811 | 0.2±0.21 | 0.4±0.09 | - | 0.5±0.03 | - | 0.3±0.42 | - | 0.8±0.01 | 0.1±0.14 | 0.6±0.09 | 0.1±0.14 | 0.5±0.01 | 0.3±0.42 | 0.2±0.29 |
| 2-methylpyridine | 819 | 0.3±0.35 | - | - | - | - | - | 0.3±0.35 | 0.2±0.24 | 0.3±0 | 0.2±0.21 | - | 0.3±0.03 | 0.2±0.24 | - |
| 2-methylpyrazine | 830 | 5.8±0.1 | 4.8±0.31 | 4.4±1.22 | 6.1±0.14 | 4.5±0.76 | 5.3±1.89 | 3.1±0.49 | 5.5±0.13 | 5.3±0.07 | 6.7±0.96 | 4.4±0.20 | 7±0.47 | 3.9±1.05 | 5.4±0.67 |
| 3-methylbutyric acid | 832 | - | - | 0.5±0.71 | - | 0.8±1.06 | - | - | - | - | - | - | - | 0.3±0.42 | - |
| furfural | 834 | 1.4±0.82 | 1.6±0.33 | 1.1±0.24 | 1.5±0.47 | 0.8±0.03 | 1.2±0.49 | 1.3±0.35 | 1.8±0.09 | 0.9±0.57 | 1.3±0.11 | 1.2±0.78 | 1.3±0.24 | 1.6±0.30 | 1.4±0.44 |
| furfuryl alcohol (syn. 2-furanmethanol) | 858 | 7.0±0.96 | 10.8±0.26 | 8±1.82 | 9.5±0.08 | 7.2±0.98 | 8.8±3.32 | 13.6±3.82 | 11±0.67 | 8.2±1.48 | 8.1±1.98 | 7.4±0.58 | 7.1±0.16 | 8.4±2.57 | 9.1±0.99 |
| acetoxyacetone (syn. 1-(acetyloxy)-2-propanone | 867 | 2.1±0.13 | 2.5±0.55 | 1.8±0.50 | 2.4±0.20 | 1.7±0.03 | 1.8±0.15 | 2.7±0.23 | 2.8±0.12 | 2.2±0.00 | 2.3±0.11 | 1.9±0.15 | 1.8±0.06 | 2.1±0.37 | 2.0±0.12 |
| 2-pentenoic acid | 873 | - | - | 0.1±0.14 | - | - | - | - | - | - | - | - | - | - | - |
| 2-heptanol | 898 | - | - | - | - | - | - | - | - | 0.2±0.21 | 0.2±0.24 | - | - | - | - |
| furfuryl formate | 904 | 0.1±0.21 | 0.3±0.06 | - | 0.4±0.08 | - | 0.2±0.21 | - | 0.5±0.00 | - | 0.4±0.06 | - | 0.2±0.21 | 0.2±0.27 | 0.3±0.03 |
| 2,6-dimethylpyrazine | 913 | 13.3±1.51 | 10.4±0.22 | 10.4±0.04 | 11±1.01 | 11.1±1.61 | 10.8±0.6 | 6.8±1.12 | 10.7±1.07 | 11±0.71 | 12.5±1.3 | 10.3±1.75 | 12.7±1.14 | 9.7±1.8 | 11.5±0.19 |
| γ-butyrolactone | 915 | - | 1.8±0.04 | 0.7±1.05 | 1.5±0.25 | 0.6±0.85 | 1.4±0.36 | 2.4±0.13 | 1.8±0.06 | 0.9±1.27 | 0.7±0.99 | 2.0±0.45 | - | 0.8±1.06 | 0.9±1.20 |
| 2-ethylpyrazine | 916 | 3.4±0.52 | 2.5±0.14 | 2.9±0.28 | 3.0±0.3 | 3.3±0.2 | 2.8±0.47 | 2.3±0.04 | 2.7±0.08 | 3.4±0.42 | 3.6±0.71 | 3.1±0.01 | 4.3±0.33 | 2.7±0.40 | 3.2±0.55 |
| 2,3-dimethylpyrazine | 923 | 1.9±0.21 | 1.5±0.11 | 1.5±0.35 | 1.4±0 | 1.7±0.23 | 1.8±0.50 | 0.9±0.33 | 1.6±0.26 | 1.6±0.07 | 1.8±0.39 | 1.5±0.27 | 1.9±0.04 | 1.3±0.29 | 1.5±0.06 |
| 2,5-hexanedione (syn. Acetonyl acetone) | 926 | - | 0.2±0.21 | - | - | - | - | - | 0.4±0.05 | - | - | - | - | - | 0.2±0.21 |
| 4-ethylpyridine | 956 | 0.7±0.12 | 0.7±0.01 | 0.5±0.04 | 0.8±0.05 | 0.6±0.09 | 0.6±0.09 | 0.6±0.00 | 0.7±0.00 | 0.6±0.00 | 0.7±0.21 | 0.7±0.09 | 0.6±0.02 | 0.7±0.02 | 0.6±0.06 |
| 1-acetoxy-2-butanone | 960 | 0.3±0.40 | 0.6±0.04 | 0.4±0.06 | 0.7±0.02 | 0.5±0.01 | 0.4±0.04 | 0.6±0.27 | 0.7±0.02 | 0.6±0.07 | 0.6±0.11 | 0.5±0.16 | 0.4±0.02 | 0.6±0.07 | 0.5±0.02 |
| 5-methylfurfural | 964 | 2.1±0.07 | 2.9±0.16 | 2.5±0.05 | 2.8±0.18 | 2.4±0.02 | 2.3±0.39 | 4.1±0.05 | 3.5±0.09 | 2.7±0.35 | 2.5±0.01 | 2.5±0.40 | 1.9±0.04 | 2.5±0.26 | 2.4±0.19 |
| phenol | 983 | - | 0.3±0.09 | - | 0.2±0.25 | - | 0.2±0.23 | - | 0.2±0.26 | - | 0.2±0.25 | 0.3±0.36 | 0.3±0.04 | 0.2±0.29 | 0.2±0.23 |
| 2-pentyl furan | 992 | - | - | 0.3±0.44 | 0.2±0.23 | 0.2±0.23 | 0.2±0.23 | 0.3±0.42 | - | - | 0.4±0.12 | - | 0.5±0.02 | - | 0.2±0.21 |
| 2-furfuryl acetate | 995 | 1.9±2.09 | 5.3±0.08 | 3.2±0.38 | 5.4±0.45 | 3.6±0.33 | 4±0.06 | 3.9±0.10 | 6.0±0.21 | 3.6±0.07 | 4.5±0.33 | 4.2±0.18 | 3.7±0.04 | 4.4±0.13 | 4.4±0.16 |
| 2-ethyl-6-methylpyrazine | 999 | 6.4±0.31 | 4.9±0.3 | 5.6±0.83 | 4.8±0.06 | 6.1±0.42 | 5.9±0.05 | 3.6±0.06 | 4.1±0.30 | 5.4±0.07 | 4.9±0.38 | 6.2±0.52 | 6±0.23 | 4.7±0.12 | 5.7±0.3 |
| 2-ethyl-5-methylpyrazine | 1002 | 3.2±0.62 | 2.8±0.37 | 3.0±0.14 | 2.7±0.18 | 3.7±0.42 | 3.4±0.10 | 2.2±0.03 | 2.4±0.33 | 2.7±0.35 | 2.6±0.23 | 3.4±0.35 | 3.2±0.08 | 2.5±0.14 | 3.2±0.36 |
| trimethylpyrazine | 1003 | 4.1±0.02 | 3.5±0.13 | 4.8±0.37 | 3.5±0.13 | 4.6±0.27 | 4.3±0.21 | 2.6±0.68 | 2.8±0.06 | 3.8±0.21 | 3.6±0.71 | 5.1±0.08 | 4.3±0.11 | 4.2±0.37 | 4.4±0.45 |
| 2-ethyl-3-methylpyrazine | 1005 | 2.3±0.01 | 2.0±0.23 | 2.1±0.64 | 1.9±0.08 | 2.6±0.40 | 2.4±0.13 | 1.1±0.80 | 1.5±0.11 | 1.7±0.57 | 1.9±0.04 | 2.5±0.29 | 2.3±0.24 | 1.8±0.17 | 2.3±0.21 |
| 3-methoxypyridine | 1006 | - | 1.6±0.24 | 0.6±0.85 | 0.6±0.36 | 1.4±0.03 | - | 1.4±0.49 | - | - | - | 1.6±0.20 | - | 1.5±0.21 | - |
| 1-(2-furanyl)-1-propanone (syn 2-furylethyl ketone) | 1008 | - | 0.2±0.32 | - | - | - | - | - | - | - | - | - | 0.2±0.24 | 0.2±0.35 | - |
| 2-propylpyrazine | 1009 | - | - | - | 0.4±0.54 | 0.3±0.42 | 0.3±0.39 | - | - | - | - | - | - | - | 0.7±0.93 |
| 2-vinyl-6-methylpyrazine | 1015 | - | 0.2±0.28 | - | 0.2±0.28 | 0.2±0.22 | 0.2±0.23 | - | - | 0.2±0.21 | 0.4±0.06 | - | 0.4±0.04 | 0.5±0.12 | 0.2±0.28 |
| 2-acetylpyrazine | 1022 | 0.4±0.58 | 0.2±0.23 | 0.5±0.68 | 0.7±0.09 | - | 0.2±0.24 | - | 0.7±0.25 | - | 0.4±0.02 | - | 0.3±0.35 | 0.4±0.58 | 0.4±0.50 |
| 5-ethyl-2-methylpyridine | 1023 | - | - | - | - | 0.2±0.34 | - | - | - | - | - | - | 0.4±0.52 | - | - |
| 3-hydroxy-2-methyl-2-cyclopenten-1-one | 1024 | - | 0.3±0.38 | - | 0.3±0.01 | 0.2±0.25 | - | 0.3±0.47 | 0.4±0.05 | 0.2±0.21 | 0.2±0.21 | 0.2±0.32 | - | 0.6±0.06 | - |
| limonene | 1032 | - | - | - | - | 0.2±0.25 | - | - | - | - | - | - | - | - | - |
| 2,3-dimethyl-2-cyclopentenone | 1038 | - | 0.5±0.24 | - | 0.6±0.01 | - | 0.3±0.35 | 0.3±0.46 | 0.2±0.28 | 0.2±0.21 | 0.3±0.35 | 0.3±0.35 | 0.2±0.23 | 0.3±0.40 | 0.3±0.35 |
| 1-ethyl-2-formylpyrrole | 1046 | - | - | - | - | - | - | - | - | 0.1±0.14 | - | - | - | - | - |
| 2-acetylpyrrole | 1062 | 1.0±0.79 | 1.4±0.11 | 1.9±0.46 | 1.1±0.01 | 1.7±0.34 | 1.2±0.33 | 2.1±0.06 | 1.2±0.01 | 1.4±0.14 | 1.0±0.11 | 1.6±0.04 | 0.9±0.04 | 1.6±0.27 | 1.2±0.04 |
| 2,3,4-trimethyl-2-cyclopenten-1-one | 1063 | - | 0.2±0.34 | - | 0.2±0.25 | - | - | - | 0.4±0.01 | - | 0.2±0.25 | - | - | 0.2±0.30 | 0.2±0.24 |
| 2-methylbenzaldehyde | 1064 | - | - | - | - | 0.2±0.25 | - | - | - | - | - | - | - | - | - |
| *cis*-linalool oxide (furanoid) | 1073 | - | 0.2±0.21 | - | 0.4±0.04 | - | - | - | 0.2±0.28 | - | 0.2±0.28 | - | - | - | - |
| 2-amino-6-methylpyridine (syn. 6-methyl-2-pyridinamine) | 1074 | 0.5±0.64 | 1±0.35 | 1±0.08 | 0.6±0.01 | 1±0.11 | 0.5±0.15 | 1.5±0.04 | 0.9±0.25 | 0.8±0.14 | 0.7±0.23 | 1.1±0.01 | 0.4±0.54 | 1.0±0.03 | 0.6±0.00 |
| 2,6-diethylpyrazine | 1083 | 6.8±1.17 | 4.8±0.25 | 6.8±0.66 | 4.7±0.21 | 7.3±0.54 | 7.2±1.10 | 4.6±0.32 | 3.6±0.45 | 6.1±0.71 | 5±0.82 | 6.3±0.08 | 6±0.63 | 5.2±0.66 | 5.9±1.07 |
| 2-ethyl-3,5-dimethyl pyrazine | 1084 | 1.4±0.08 | 1.3±0.24 | 1.4±0.22 | 1.1±0.09 | 1.5±0.03 | 1.6±0.03 | 1.3±0.29 | - | 1.5±0.14 | 1.3±0.08 | 1.6±0.01 | 1.3±0.09 | 1.4±0.1 | 1.4±0.12 |
| 2-furfurylfuran | 1085 | - | - | - | - | - | - | - | 1.2±0.11 | - | - | - | - | - | - |
| 2,3-diethylpyrazine | 1086 | 2.2±0.18 | 1.7±0.48 | 2.7±0.28 | 1.4±0.03 | 2.6±0.13 | 1.8±0.01 | 1.5±0.07 | 1.0±0.30 | 1.8±0.00 | 1.4±0.11 | 2.2±0.21 | 1.6±0.02 | 2.0±0.15 | 1.8±0.20 |
| *o*-guaiacol | 1090 | 1.7±0.18 | 1.3±0.28 | 1.7±0.28 | 1.0±0.06 | 1.4±0.13 | 1.1±0.11 | 2.6±0.39 | 1.3±0.01 | 1.5±0.14 | 1.3±0.25 | 1.6±0.14 | 1.1±0.06 | 2.1±0.28 | 1.5±0.06 |
| 3-ethyl-2-hydroxy-2-cyclopenten-1-one | 1091 | - | 0.5±0.13 | - | - | 0.4±0.49 | 0.5±0.06 | 0.7±0.08 | 0.6±0.01 | 0.3±0.35 | 0.2±0.33 | 0.7±0.08 | 0.5±0.06 | 0.7±0.1 | 0.5±0.02 |
| 2-methyl-3-propylpyrazine | 1092 | 0.6±0.11 | - | 0.6±0.04 | 0.5±0.03 | 0.3±0.39 | - | - | - | 0.3±0.42 | 0.3±0.42 | - | - | - | - |
| nonanal | 1104 | - | - | - | - | - | 0.2±0.21 | - | - | - | - | - | - | - | 0.2±0.23 |
| *iso*propenylpyrazine | 1107 | 1.5±0.16 | 1.0±0.13 | 1.5±0.08 | 1.0±0.16 | 1.2±0.03 | 1.2±0.28 | 1.2±0.21 | 0.8±0.08 | 1.2±0.00 | 1±0.08 | 1.4±0.23 | 1.2±0.01 | 1.1±0.01 | 1.2±0.05 |
| maltol | 1111 | 0.7±0.10 | 1.6±0.42 | 0.9±0.26 | 1.5±0.03 | 0.5±0.04 | 1.0±0.66 | 1.3±0.07 | 1.4±0.07 | 0.5±0.14 | 1±0.06 | 1.2±0.03 | 0.7±0.04 | 1.1±0.19 | 1±0.17 |
| phenylethyl alcohol | 1112 | - | - | - | - | - | - | - | - | - | - | - | - | 1.1±0.23 | - |
| 4-methyl-2-aminopyridine | 1113 | - | - | - | - | - | - | 0.4±0.56 | - | - | - | 0.2±0.33 | - | 0.2±0.24 | - |
| methyl nicotinate | 1138 | - | - | - | 0.4±0.04 | 0.2±0.25 | - | - | 0.2±0.23 | - | - | - | - | 0.2±0.28 | 0.2±0.21 |
| 5H-5-methyl-6,7-dihydrocyclopentapyrazine | 1141 | 0.5±0.64 | 0.5±0.04 | 1.1±0.09 | 0.7±0.04 | 0.9±0.06 | 0.7±0.06 | 0.8±0.08 | 0.5±0.06 | 1±0.28 | 0.7±0.05 | 1±0.26 | 0.8±0.02 | 0.7±0.44 | 0.8±0.09 |
| 2,3-diethyl-5-methylpyrazine | 1157 | 0.2±0.32 | 0.2±0.21 | - | 0.2±0.24 | 0.5±0.12 | 0.5±0.01 | - | - | 0.5±0.07 | 0.2±0.23 | - | 0.2±0.27 | - | 0.4±0.04 |
| 3,5-diethyl-2-methylpyrazine | 1158 | 1.3±0.17 | 1.1±0.37 | 1.4±0.06 | 0.9±0.19 | 1.7±0.26 | 1.4±0.10 | 0.9±0.21 | 0.7±0.17 | 1.5±0.07 | 1.1±0.15 | 1.3±0.29 | 1.1±0.23 | 1±0.08 | 1.2±0.11 |
| 2,3,5-trimethyl-6-ethylpyrazine | 1165 | 0.3±0.42 | 0.2±0.26 | 0.6±0.06 | 0.2±0.27 | 0.2±0.23 | 0.5±0.04 | - | - | 0.6±0.07 | 0.2±0.27 | 0.3±0.37 | 0.2±0.34 | 0.2±0.27 | 0.2±0.32 |
| *endo*-borneol | 1166 | - | 0.2±0.23 | - | - | - | - | - | - | - | - | - | - | - | - |
| 2-furfuryl-5-methylfuran | 1184 | - | 0.2±0.33 | - | 0.2±0.21 | - | 0.2±0.21 | - | 0.5±0.02 | - | 0.2±0.28 | 0.3±0.35 | 0.2±0.21 | 0.4±0.05 | 0.2±0.21 |
| 1-furfurylpyrrole | 1185 | 0.3±0.49 | 0.8±0.16 | 0.6±0.82 | 0.7±0.03 | 0.7±0.08 | 0.9±0.13 | 1.2±0.30 | 0.9±0.05 | 0.8±0.49 | 0.8±0.14 | 0.8±0.21 | 0.7±0.02 | 0.9±0.19 | 0.9±0.02 |
| 2-butanoyl-5-methylfuran | 1188 | - | - | - | - | - | - | - | - | 0.2±0.21 | - | - | - | 0.4±0.11 | - |
| 2-methyl-5[(*Z*)-1-propenyl]pyrazine | 1192 | 1.0±0.09 | 0.6±0.06 | 1.3±0.14 | 0.5±0.03 | 0.8±0.23 | 0.7±0.09 | 0.5±0.64 | - | 0.8±0.07 | 0.5±0.04 | 0.9±0.08 | 0.7±0.02 | 0.8±0.01 | 0.8±0.06 |
| methyl salicylate | 1193 | - | - | - | - | - | - | 0.4±0.54 | - | - | - | - | - | 0.5±0.05 | - |
| decanal | 1204 | - | - | - | - | 0.2±0.21 | - | - | - | - | - | - | - | - | - |
| verbenone | 1205 | 0.3±0.36 | - | 0.3±0.44 | 0.2±0.24 | 0.2±0.28 | 0.2±0.27 | 0.3±0.46 | - | 0.5±0 | - | 0.3±0.45 | 0.2±0.26 | 0.2±0.28 | 0.2±0.26 |
| 2,4-dimethylacetophenone | 1220 | - | - | - | - | - | 0.3±0.35 | 0.4±0.63 | - | - | - | - | - | - | - |
| 2-vinylbenzofuran | 1221 | - | - | - | - | 0.4±0.57 | - | - | - | - | - | - | 0.2±0.21 | - | - |
| 2,5-dimethyl-6,7-dihydro-(5H)-cyclopentapyrazine | 1222 | - | - | 1±0.08 | - | 0.5±0.66 | - | - | - | 0.9±0 | - | 0.9±0.31 | - | 0.6±0.05 | - |
| furfuryl 3-methylbutanoate | 1223 | - | 0.3±0.43 | - | 0.3±0.47 | 0.2±0.21 | 0.5±0.26 | - | 0.3±0.38 | - | 0.3±0.44 | 0.2±0.28 | 0.6±0.16 | 0.2±0.28 | 0.4±0.49 |
| phenyl butyrate | 1245 | - | - | - | - | 0.2±0.28 | - | - | - | - | - | - | - | - | - |
| 2-methyl-6-*iso*pentylpyrazine | 1249 | 0.3±0.38 | - | 0.5±0.02 | - | 0.5±0.21 | - | 0.3±0.42 | - | 0.6±0.07 | - | 0.4±0.51 | - | 0.5±0.13 | - |
| *p*-ethylguaiacol | 1280 | 2.7±0.08 | 1.4±0.01 | 2.4±0.59 | 0.9±0.01 | 1.8±0.65 | 1.4±0.20 | 2.7±0.28 | 1.1±0.21 | 2.1±0.14 | 1.3±0.08 | 2.5±0.19 | 1.7±0.23 | 2.4±0.38 | 1.7±0.21 |
| (E)-anethole | 1283 | - | 0.2±0.23 | - | - | 0.2±0.29 | 0.2±0.24 | - | - | 0.3±0.35 | 0.2±0.23 | - | - | - | 0.2±0.23 |
| difurfuryl ether | 1302 | 0.3±0.36 | 0.5±0.04 | - | 0.5±0.06 | 0.5±0.04 | 0.5±0.00 | 0.4±0.62 | 0.5±0.12 | 0.6±0.21 | 0.4±0.01 | 0.8±0.03 | 0.4±0.13 | 0.7±0.21 | 0.5±0.03 |
| *p*-vinylguaiacol | 1314 | 6.4±0.88 | 3.3±0.55 | 7.2±2.26 | 3.0±0.01 | 5.1±1.65 | 4.1±0.54 | 8.1±0.29 | 3.5±0.06 | 5.9±0.21 | 4.6±0.64 | 4.7±0.08 | 4.4±0.89 | 6.3±1.58 | 4.8±0.72 |
| 2,5-dimehtyl-3-*iso*pentylpyrazine | 1315 | 0.5±0.11 | - | 0.6±0.01 | - | 0.6±0.08 | - | - | - | 0.7±0.07 | - | 0.3±0.43 | - | 0.4±0.01 | 0.2±0.21 |
| 4-vinylveratrole | 1367 | - | - | - | - | - | - | 2.3±0.28 | 0.3±0.47 | 0.5±0 | - | - | - | 0.4±0.62 | 0.2±0.33 |
| (E)-β-damascenone | 1382 | - | 0.2±0.28 | 0.6±0.01 | - | - | 0.3±0.36 | 0.3±0.47 | 0.2±0.23 | 0.4±0.49 | 0.2±0.35 | 0.3±0.42 | 0.3±0.39 | - | 0.2±0.31 |
| skatole (syn. 3-methylindole) | 1383 | - | - | - | - | - | - | - | - | - | - | 0.5±0.64 | - | - | - |
|  |  |  |  |  |  |  |  |  |  |  |  |  |  |  |  |
| Acids/esters | | 1.1±0.18 | 4.6±0.07 | 1.8±0.98 | 3.3±0.14 | 2.1±1.49 | 3.5±1.35 | 1.7±0.11 | 3.7±0.05 | 1.1±0.28 | 2.6±0.58 | 1.3±0.37 | 2.4±0.20 | 1.2±0.53 | 2.8±0.03 |
| Terpenes | | 0.3±0.36 | 0.3±0.01 | 0.3±0.44 | 0.6±0.28 | 0.4±0.03 | 0.2±0.27 | 0.3±0.46 | 0.2±0.28 | 0.5±0.00 | 0.2±0.28 | 0.3±0.45 | 0.2±0.26 | 0.2±0.28 | 0.2±0.26 |
| Aldehydes-ketones | | 2.5±0.40 | 5.4±1.22 | 2.2±0.56 | 4.6±0.49 | 3.1±0.16 | 3.5±0.30 | 4.7±1.34 | 6.2±0.04 | 3.8±1.34 | 4.2±1.08 | 3.6±0.61 | 3.3±0.25 | 4.3±1.17 | 4.1±0.73 |
| Pyrazines | | 57.3±3.06 | 44.3±2.47 | 54.7±3.28 | 46.9±0.75 | 56.7±4.45 | 53.3±2.93 | 33.4±0.88 | 39±0.94 | 52.2±1.06 | 50.7±1.01 | 52.9±0.16 | 55.9±2.4 | 45.6±3.13 | 52.3±0.03 |
| Pyridines-piperidines | | 5.9±1.50 | 8.8±1.44 | 4.5±0.50 | 10.6±0.5 | 5.5±1.42 | 5.6±3.05 | 7.3±0.03 | 9.7±1.19 | 5.6±0.00 | 9.3±2.33 | 6.4±0.66 | 8.4±0.37 | 7.8±2.35 | 5.9±1.32 |
| Pyrroles | | 1.3±1.28 | 2.2±0.05 | 2.5±0.36 | 1.8±0.04 | 2.3±0.26 | 2.1±0.46 | 3.3±0.24 | 2.1±0.06 | 2.3±0.49 | 1.8±0.25 | 2.3±0.25 | 1.6±0.06 | 2.5±0.46 | 2.1±0.06 |
| Phenols | | 10.7±0.98 | 6.3±0.75 | 11.3±3.13 | 5.0±0.19 | 8.4±2.43 | 6.8±0.63 | 13.8±0.15 | 6.1±0.42 | 9.5±0.21 | 7.5±0.74 | 9.0±0.77 | 7.6±1.22 | 11.5±2.00 | 8.1±0.76 |
| Alcohols | | - | 0.2±0.21 | 0.1±0.14 | - | 0.2±0.21 | - | 0.4±0.57 | - | 0.2±0.21 | 0.2±0.24 | - | - | 1.1±0.23 | - |
| Furans | | 12.9±2.37 | 24.5±1.36 | 15.8±0.23 | 22.7±0.83 | 15.9±1.96 | 19.6±3.03 | 25.9±3.8 | 27.7±0.36 | 17±2.76 | 19.4±3.01 | 18.6±0.12 | 16.6±0.01 | 20.0±2.86 | 19.8±1.32 |
| Apocarotenes | | - | 0.2±0.28 | 0.6±0.01 | - | - | 0.3±0.36 | 0.3±0.47 | 0.2±0.23 | 0.4±0.49 | 0.2±0.35 | 0.3±0.42 | 0.3±0.39 | - | 0.2±0.31 |
| Others | | 0.7±0.1 | 1.8±0.18 | 0.9±0.26 | 2±0.71 | 0.7±0.25 | 1.7±0.40 | 4.0±0.42 | 1.7±0.4 | 1.3±0.49 | 1.1±0.17 | 1.7±0.61 | 0.7±0.04 | 1.5±0.43 | 1.7±1.15 |
|  |  |  |  |  |  |  |  |  |  |  |  |  |  |  |  |
| Total identified (%) | | 92.6±2.14 | 98.3±0.01 | 94.6±0.87 | 97.6±1.44 | 95.3±0.42 | 96.4±1.04 | 95.1±0.53 | 96.6±0.59 | 93.5±1.27 | 97.2±0.32 | 96.4±2.38 | 96.8±0.11 | 95.7±1.27 | 97.1±1.58 |
| ^a^ Linear retention indices on a HP5-MS capillary column; ^b^ Not detected. Legend: W, whole; G, ground. | | | | | | | | | | | | | | | |

**Table S6**. Complete headspace compositions of the whole (W) and ground (G) commercial blend samples.

| **Compound** | **l.r.i.^a^** | **Relative abundance (%) ± SD** | | | | | | | |
| --- | --- | --- | --- | --- | --- | --- | --- | --- | --- |
|  |  | **GC_W** | **GC_G** | **GD_W** | **GD_G** | **PH_W** | **PH_G** | **PV_W** | **PV_G** |
| acetic acid | 603 | 0.9±0.45 | 3.0±0.84 | 0.7±0.21 | 2.2±0.22 | 1.3±0.01 | 3.6±0.01 | 0.4±0.12 | 3.3±1.09 |
| 2-butanone | 604 | -^b^ | 0.2±0.28 | - | - | - | - | 0.1±0.07 | - |
| ethyl acetate | 616 | 0.5±0.29 | 0.3±0.42 | - | - | - | - | - | - |
| 3-methyl furan | 646 | - | - | - | - | - | - | 0.2±0.26 | - |
| *iso*valeraldehyde (syn. 3-methylbutanal) | 653 | - | 0.4±0.33 | - | 0.1±0.07 | - | 0.1±0.07 | 0.3±0.24 | - |
| 1-hydroxy-2-propanone (syn. Acetol) | 656 | - | 0.2±0.21 | 0.1±0.14 | 0.2±0.28 | 0.1±0.14 | 0.5±0.06 | 0.1±0.14 | 0.4±0.10 |
| 2-methylbutanal | 659 | - | 0.2±0.28 | - | 0.4±0.06 | 0.1±0.07 | 0.1±0.14 | - | - |
| 3-methyl-2-butanol | 673 | - | - | - | 0.3±0.42 | - | - | 0.3±0.40 | - |
| 2,3-pentanedione | 699 | - | 0.1±0.14 | - | 0.1±0.07 | 0.2±0.15 | 0.2±0.11 | 0.3±0.08 | - |
| 3-hydroxy-2-butanone (syn. Acetoin) | 707 | - | 0.2±0.21 | 0.1±0.07 | 0.3±0.06 | 0.3±0.08 | 0.3±0.03 | 0.5±0.25 | 0.1±0.14 |
| pyrazine | 737 | 0.2±0.22 | 0.6±0.04 | 0.1±0.14 | 0.6±0.09 | 0.4±0.12 | 0.5±0.08 | 0.3±0.42 | 0.3±0.35 |
| pyridine | 747 | 3.7±1.82 | 11.5±0.32 | 5.8±1.58 | 9.3±1.48 | 9.6±0.88 | 10.5±0.81 | 11.9±0.23 | 7.6±4.43 |
| 1-methylpiperidine | 777 | 0.3±0.10 | 0.5±0.21 | 0.2±0.28 | 0.4±0.08 | 1.1±0.09 | 0.5±0.11 | 0.9±0.32 | 0.7±0.03 |
| 1,3-butanediol | 788 | 0.4±0.08 | 0.1±0.07 | 0.1±0.14 | 0.2±0.01 | 0.4±0.13 | 0.1±0.14 | 0.3±0.35 | 0.3±0.05 |
| 2,3-butanediol | 790 | 0.5±0.37 | 0.1±0.14 | 0.1±0.07 | 0.1±0.14 | 0.3±0.11 | 0.1±0.14 | 0.2±0.28 | 0.1±0.14 |
| 3,5-dimethylisoxazole | 801 | 0.3±0.35 | 1.0±0.08 | 1.1±0.06 | 0.7±0.26 | 2.3±0.15 | 0.9±0.25 | 1.9±0.27 | 1.0±0.23 |
| hexanal | 802 | - | 0.1±0.14 | - | 0.2±0.28 | - | 0.3±0.35 | - | 0.2±0.21 |
| 3-hexanol | 806 | - | - | - | - | 0.3±0.10 | - | - | - |
| dihydro-2-methyl-3(2H)-furanone | 811 | 0.2±0.3 | 0.9±0.17 | 0.4±0.49 | 0.8±0.43 | 1.2±0.30 | 0.8±0.28 | 1.1±0.05 | 0.4±0.49 |
| 2-methylpyridine | 819 | 0.3±0.05 | 0.2±0.21 | 0.3±0.35 | 0.4±0.21 | 0.4±0.06 | 0.2±0.21 | 0.5±0.05 | 0.2±0.28 |
| 2-methylpyrazine | 830 | 5.0±0.91 | 6.2±0.28 | 4.1±1.27 | 6.9±0.44 | 6.1±0.07 | 5.4±0.32 | 6.8±0.65 | 4.7±2.24 |
| furfural | 834 | 1.8±0.86 | 1.6±0.10 | 1.4±0.45 | 1.7±0.42 | 1.4±0.15 | 2.0±0.30 | 1.2±0.06 | 1.4±0.53 |
| ethyl-2-methyl butyrate | 847 | 0.2±0.22 | - | - | - | - | - | - | - |
| furfuryl alcohol (syn. 2-furanmethanol) | 858 | 9.9±2.36 | 8.9±0.02 | 11.0±0.64 | 7.1±0.30 | 8.4±0.82 | 9.2±0.83 | 6.5±0.88 | 10.4±2.21 |
| 3-methylpyridine | 866 | 0.2±0.21 | 0.2±0.21 | 0.3±0.08 | 0.2±0.03 | 0.3±0.06 | 0.1±0.14 | 0.3±0.04 | 0.2±0.21 |
| acetoxyacetone (syn. 1-(acetyloxy)-2-propanone | 867 | 2.3±0.08 | 2.9±0.15 | 3.2±0.06 | 2.3±0.24 | 3.3±0.03 | 2.7±0.20 | 2.8±0.3 | 2.6±0.07 |
| furfuryl formate | 904 | 0.2±0.21 | 0.6±0.04 | 0.4±0.02 | 0.4±0.13 | 0.6±0.04 | 0.5±0.04 | 0.5±0.04 | 0.4±0.04 |
| (*E,E*)-2,4-hexadienal | 911 | - | - | 0.2±0.21 | 0.2±0.21 | 0.2±0.21 | 0.1±0.14 | 0.2±0.21 | 0.1±0.14 |
| 2,6-dimethylpyrazine | 913 | 9.2±0.41 | 10.0±0.63 | 11.1±0.76 | 10.8±0.16 | 11.0±0.22 | 9.7±0.08 | 11.4±0.2 | 10.5±0.45 |
| γ-butyrolactone | 915 | 1.8±0.67 | 1.9±0.71 | 1.9±0.16 | 1.2±0.55 | 1.4±0.28 | 1.4±0.80 | 1.5±0.39 | 1.7±0.75 |
| 2-ethylpyrazine | 916 | 3.1±0.09 | 3.2±0.26 | 3.1±0.04 | 3.8±0.29 | 3.2±0.36 | 2.8±0.36 | 3.7±0.51 | 2.8±0.88 |
| 2,3-dimethylpyrazine | 923 | 1.6±0.16 | 1.6±0.43 | 1.9±0.35 | 2.0±0.16 | 1.7±0.33 | 1.6±0.07 | 2.1±0.04 | 1.4±0.51 |
| 2,5-hexanedione (syn. Acetonyl acetone) | 926 | 0.1±0.14 | 0.5±0.08 | 0.5±0.12 | - | 0.3±0.40 | 0.2±0.25 | 0.3±0.38 | - |
| 2,4-dimethylpyridine | 927 | - | 0.2±0.23 | - | 0.1±0.20 | 0.2±0.25 | - | 0.2±0.24 | - |
| 3-methyl-2,5-furandione | 944 | 0.1±0.14 | 0.2±0.25 | 0.3±0.06 | 0.1±0.17 | 0.2±0.30 | 0.2±0.25 | 0.2±0.25 | 0.2±0.24 |
| 4-ethylpyridine | 956 | 0.7±0.03 | 1.0±0.02 | 1.1±0.06 | 0.8±0.04 | 1.1±0.15 | 1.0±0.13 | 1.2±0.07 | 1.1±0.04 |
| 1-acetoxy-2-butanone | 960 | 0.7±0.06 | 0.9±0.08 | 0.4±0.63 | 0.6±0.10 | 1.1±0.16 | 0.9±0.13 | 0.8±0.10 | 0.4±0.59 |
| 5-methylfurfural | 964 | 2.8±0.29 | 3.1±0.02 | 3.2±0.00 | 2.3±0.29 | 3.0±0.40 | 3.2±0.02 | 2.1±0.01 | 2.6±0.46 |
| phenol | 983 | 0.2±0.21 | - | - | 0.2±0.25 | 0.2±0.23 | - | 0.2±0.32 | - |
| 2-pentyl furan | 992 | 0.4±0.52 | 0.2±0.25 | - | - | - | 0.2±0.25 | 0.2±0.23 | 0.2±0.27 |
| 2-furfuryl acetate | 995 | 4.5±0.08 | 7.8±0.16 | 7.8±0.52 | 5.5±0.94 | 7.9±0.54 | 7.2±0.13 | 6.7±0.25 | 7.1±0.12 |
| 2-ethyl-6-methylpyrazine | 999 | 5.0±0.40 | 4.0±0.18 | 4.3±0.05 | 4.8±0.25 | 3.5±0.25 | 3.7±0.05 | 4.3±0.21 | 4.9±0.35 |
| 2-ethyl-5-methylpyrazine | 1002 | 2.8±0.07 | 2.3±0.08 | 2.3±0.11 | 2.9±0.13 | 2.0±0.07 | 2.1±0.05 | 2.3±0.34 | 2.7±0.13 |
| trimethylpyrazine | 1003 | 4.3±0.72 | 2.9±0.11 | 3.5±0.16 | 3.4±0.18 | 2.9±0.34 | 3.0±0.16 | 3.3±0.17 | 3.9±0.06 |
| 2-ethyl-3-methylpyrazine | 1005 | 2.0±0.15 | 1.5±0.02 | 1.6±0.13 | 1.9±0.11 | 1.3±0.08 | 1.2±0.33 | 1.6±0.01 | 1.9±0.02 |
| 3-methoxypyridine | 1006 | 1.6±0.01 | 1.3±0.23 | 1.0±1.44 | - | 1.2±0.25 | 0.8±1.10 | 0.4±0.59 | 0.9±1.22 |
| 1-(2-furanyl)-1-propanone (syn 2-furylethyl ketone) | 1008 | 0.2±0.23 | 0.2±0.28 | 0.2±0.33 | 0.2±0.23 | 0.2±0.28 | 0.2±0.33 | 0.2±0.25 | 0.2±0.27 |
| 2-propylpyrazine | 1009 | - | - | - | - | - | - | 0.3±0.37 | - |
| 2-vinyl-6-methylpyrazine | 1015 | 0.4±0.05 | 0.4±0.01 | 0.5±0.06 | 0.4±0.06 | 0.4±0.08 | 0.4±0.01 | 0.4±0.04 | 0.2±0.30 |
| 2-acetylpyrazine | 1022 | - | - | 0.4±0.58 | 0.4±0.50 | 0.3±0.43 | - | - | - |
| 5-ethyl-2-methylpyridine | 1023 | 0.8±0.11 | 0.8±0.11 | - | - | - | - | - | - |
| 3-hydroxy-2-methyl-2-cyclopenten-1-one | 1024 | - | - | 0.2±0.25 | - | 0.2±0.25 | 0.2±0.25 | - | - |
| limonene | 1032 | 0.4±0.10 | - | 0.3±0.42 | - | - | 0.1±0.18 | - | - |
| benzyl alcohol | 1035 | 0.3±0.06 | - | - | - | - | - | - | - |
| 2,3-dimethyl-2-cyclopentenone | 1038 | 0.2±0.21 | 0.6±0.16 | 0.8±0.02 | 0.4±0.11 | 0.6±0.25 | 0.6±0.18 | 0.5±0.16 | 0.4±0.52 |
| 1-ethyl-2-formylpyrrole | 1046 | - | - | 0.2±0.24 | - | - | - | - | - |
| 2-acetylpyrrole | 1062 | 1.6±0.25 | 1.1±0.01 | 1.5±0.21 | 1.1±0.04 | 0.9±0.35 | 1.2±0.04 | 1.0±0.05 | 1.2±0.45 |
| 2,3,4-trimethyl-2-cyclopenten-1-one | 1063 | - | 0.2±0.25 | 0.2±0.30 | 0.3±0.02 | 0.4±0.04 | 0.4±0.02 | 0.3±0.04 | 0.4±0.07 |
| 2-methylbenzaldehyde | 1064 | 0.2±0.25 | - | - | 0.1±0.17 | - | - | - | - |
| *cis*-linalool oxide (furanoid) | 1073 | - | 0.5±0.19 | 0.6±0.01 | 0.1±0.14 | 0.4±0.03 | 0.5±0.07 | 0.2±0.28 | 0.2±0.28 |
| 2-amino-6-methylpyridine (syn. 6-methyl-2-pyridinamine) | 1074 | 1.1±0.22 | 0.6±0.03 | 0.8±0.27 | 0.8±0.10 | 0.8±0.06 | 0.7±0.01 | 0.8±0.30 | 0.9±0.21 |
| 2,6-diethylpyrazine | 1083 | 5.0±0.71 | 3.0±0.33 | 3.6±0.12 | 4.2±0.21 | 2.7±0.52 | 3.0±0.17 | 3.4±0.06 | 4.2±0.10 |
| 2-furfurylfuran | 1085 | 1.4±0.13 | 1.2±0.04 | 1.3±0.11 | 1.2±0.04 | 1.1±0.18 | 1.2±0.04 | 1.0±0.19 | 1.4±0.13 |
| 2,3-diethylpyrazine | 1086 | 1.8±0.18 | 0.8±0.29 | 1.1±0.22 | 1.1±0.30 | 0.6±0.17 | 0.7±0.08 | 0.7±0.08 | 1.0±0.10 |
| *o*-guaiacol | 1090 | 2.2±0.51 | 1.0±0.06 | 1.5±0.01 | 1.3±0.05 | 0.9±0.23 | 1.0±0.02 | 0.9±0.28 | 1.4±0.14 |
| 3-ethyl-2-hydroxy-2-cyclopenten-1-one | 1091 | 0.5±0.03 | 0.5±0.04 | 0.6±0.07 | - | 0.8±0.28 | 0.6±0.05 | 0.5±0.02 | 0.5±0.01 |
| 2-methyl-3-propylpyrazine | 1092 | - | - | - | 0.4±0.11 | - | - | - | - |
| 4,4-dimethyl-2-cyclohexen-1-one | 1102 | - | 0.2±0.23 | 0.2±0.24 | 0.1±0.15 | - | - | 0.2±0.22 | 0.2±0.30 |
| *iso*propenylpyrazine | 1107 | 1.1±0.23 | 0.9±0.01 | 1.1±0.10 | 0.9±0.16 | 0.9±0.11 | 0.9±0.04 | 1.0±0.04 | 1.1±0.18 |
| phenylethyl alcohol | 1112 | 1.1±0.12 | 0.5±0.25 | 0.9±0.30 | 0.6±0.13 | 0.6±0.23 | 0.4±0.60 | 0.4±0.55 | 0.7±0.45 |
| methyl nicotinate | 1138 | - | - | 0.2±0.28 | 0.1±0.14 | 0.2±0.22 | 0.2±0.24 | 0.2±0.30 | - |
| 5H-5-methyl-6,7-dihydrocyclopentapyrazine | 1141 | 0.8±0.25 | 0.5±0.05 | 0.5±0.06 | 0.6±0.10 | 0.2±0.27 | 0.3±0.35 | 0.3±0.37 | 0.3±0.45 |
| 2,3-diethyl-5-methylpyrazine | 1157 | 0.2±0.27 | - | - | 0.2±0.24 | - | - | - | - |
| 3,5-diethyl-2-methylpyrazine | 1158 | 0.6±0.91 | 0.4±0.01 | 0.7±0.06 | 0.8±0.30 | 0.5±0.04 | 0.6±0.24 | 0.7±0.31 | 0.5±0.15 |
| 2,3,5-trimethyl-6-ethylpyrazine | 1165 | 0.2±0.26 | - | - | 0.2±0.26 | - | 0.1±0.18 | - | - |
| 2-furfuryl-5-methylfuran | 1184 | 0.5±0.23 | 0.5±0.15 | 0.8±0.06 | 0.4±0.08 | 0.5±0.16 | 0.7±0.03 | 0.5±0.07 | 0.6±0.11 |
| 1-furfurylpyrrole | 1185 | 1.4±0.21 | 0.8±0.04 | 1.1±0.18 | 0.9±0.08 | 0.9±0.16 | 1.0±0.08 | 0.9±0.20 | 0.9±0.18 |
| 2-butanoyl-5-methylfuran | 1188 | 0.2±0.3 | - | - | 0.2±0.23 | - | 0.3±0.44 | - | 0.2±0.34 |
| 2-methyl-5[(*Z*)-1-propenyl]pyrazine | 1192 | 0.8±0.01 | 0.2±0.22 | 0.5±0.11 | 0.5±0.25 | 0.2±0.21 | 0.4±0.20 | 0.2±0.34 | 0.2±0.35 |
| methyl salicylate | 1193 | 0.2±0.23 | - | - | 0.1±0.16 | - | - | - | - |
| verbenone | 1205 | 0.2±0.23 | - | - | 0.1±0.18 | - | - | - | - |
| 2-vinylbenzofuran | 1221 | - | - | - | - | - | 0.1±0.14 | - | - |
| 2,5-dimethyl-6,7-dihydro-(5H)-cyclopentapyrazine | 1222 | 0.8±0.04 | - | - | - | - | - | - | - |
| furfuryl 3-methylbutanoate | 1223 | - | 0.2±0.26 | 0.5±0.31 | 0.3±0.42 | 0.4±0.01 | 0.4±0.15 | 0.3±0.35 | 0.3±0.44 |
| 2-methyl-6-*iso*pentylpyrazine | 1249 | 0.2±0.24 | - | - | - | - | - | - | - |
| *p*-ethylguaiacol | 1280 | 2.0±0.33 | 0.5±0.08 | 0.9±0.03 | 1.1±0.30 | 0.5±0.01 | 0.6±0.13 | 0.8±0.23 | 1.1±0.37 |
| (*E*)-anethole | 1283 | 0.2±0.23 | - | - | - | - | 0.1±0.20 | - | 0.2±0.21 |
| difurfuryl ether | 1302 | 0.7±0.04 | 0.5±0.13 | 0.9±0.17 | 0.5±0.00 | 0.7±0.04 | 0.7±0.12 | 0.5±0.21 | 0.5±0.11 |
| *p*-vinylguaiacol | 1314 | 3.1±0.49 | 0.9±0.11 | 1.6±0.16 | 2.2±0.57 | 0.9±0.01 | 1.2±0.05 | 1.1±0.30 | 1.6±0.35 |
| 4-vinylveratrole | 1367 | 0.3±0.37 | - | - | 0.1±0.16 | - | - | - | - |
| (*E*)-β-damascenone | 1382 | 0.2±0.26 | - | - | 0.2±0.23 | - | 0.1±0.18 | - | 0.2±0.21 |
|  |  |  |  |  |  |  |  |  |  |
| Acids/esters | | 1.6±0.95 | 3.3±0.42 | 0.7±0.21 | 2.2±0.22 | 1.3±0.01 | 3.6±0.01 | 0.4±0.12 | 3.3±1.09 |
| Terpenes | | 0.6±0.13 | 0.5±0.19 | 0.9±0.41 | 0.2±0.04 | 0.4±0.03 | 0.6±0.11 | 0.2±0.28 | 0.2±0.28 |
| Aldehydes-ketones | | 3.9±0.27 | 7.1±0.49 | 6.3±1.01 | 5.2±0.85 | 7.4±0.22 | 7.0±0.04 | 6.6±0.72 | 5.2±0.96 |
| Pyrazines | | 44.9±0.33 | 38.5±0.23 | 40.3±1.04 | 46.6±2.32 | 38.0±1.16 | 36.2±0.76 | 42.9±0.73 | 40.5±3.36 |
| Pyridines-piperidines | | 8.6±1.62 | 16.1±0.04 | 9.5±0.22 | 12.1±1.33 | 14.9±1.42 | 14.0±0.21 | 16.4±0.94 | 11.5±3.49 |
| Pyrroles | | 3.0±0.47 | 1.9±0.03 | 2.8±0.26 | 2.0±0.12 | 1.7±0.51 | 2.3±0.05 | 1.9±0.15 | 2.2±0.64 |
| Phenols | | 7.6±1.32 | 2.3±0.13 | 4.0±0.19 | 4.9±1.34 | 2.5±0.01 | 2.8±0.16 | 3.0±0.57 | 4.1±0.86 |
| Alcohols | | 1.9±0.34 | 0.6±0.04 | 1.1±0.08 | 1.2±0.42 | 1.5±0.56 | 0.6±0.32 | 1.1±0.32 | 1.2±0.36 |
| Furans | | 24.7±1.96 | 27.6±0.04 | 30.1±0.06 | 21.9±2.14 | 27.0±1.73 | 28.2±1.08 | 22.5±0.35 | 27.5±2.64 |
| Apocarotenes | | 0.2±0.26 | - | - | 0.2±0.23 | - | 0.1±0.18 | - | 0.2±0.21 |
| Others | | 1.0±0.30 | 1.0±0.08 | 1.1±0.06 | 0.8±0.10 | 2.3±0.15 | 1.1±0.05 | 1.9±0.27 | 1.2±0.02 |
|  |  |  |  |  |  |  |  |  |  |
| Total identified (%) | | 98.1±0.06 | 98.8±0.03 | 96.8±0.90 | 97.3±0.81 | 96.9±1.37 | 96.5±0.53 | 96.8±0.88 | 96.9±0.40 |
| ^a^ Linear retention indices on a HP5-MS capillary column; ^b^ Not detected. Legend: W, whole; G, ground. | | | | | | | | | |

**Table S7.** Mean liking scores of coffee samples

| **Sample ID** | **Overall liking** |
| --- | --- |
| A1 | 41.85±14.49 ^de^ |
| A2 | 47.48±14.17 ^abcd^ |
| A3 | 49.67±14.89 ^ab^ |
| A4 | 42.29±15.74 ^de^ |
| A5 | 46.88±15.26 ^abcde^ |
| A6 | 42.84±13.68 ^cde^ |
| A7 | 47.17±15.96 ^abcde^ |
| A8 | 45.43±14.24 ^abcde^ |
| A9 | 41.61±15.94 ^e^ |
| GC | 41.83±14.30 ^de^ |
| GD | 50.31±15.29 ^a^ |
| PH | 44.14±14.65 ^bcde^ |
| PV | 46.40±15.25 ^abcde^ |
| R1 | 47.01±16.87 ^abcde^ |
| R2 | 48.93±14.90 ^ab^ |
| R3 | 48.12±15.74 ^abc^ |
| R4 | 48.79±15.46 ^ab^ |
| R5 | 48.36±17.04 ^abc^ |
| R6 | 49.08±16.19 ^ab^ |
| R7 | 49.19±16.34 ^ab^ |

Data are expressed as mean ± standard deviation. Values with different letter are statistically different (p<0.05), following pairwise comparison by Tukey’s HSD test.

| **Attribute** | **Sample** | | | | | | | | | | | | | | | | | | | |
| --- | --- | --- | --- | --- | --- | --- | --- | --- | --- | --- | --- | --- | --- | --- | --- | --- | --- | --- | --- | --- |
|  | **A1** | **A2** | **A3** | **A4** | **A5** | **A6** | **A7** | **A8** | **A9** | **GC** | **GD** | **PH** | **PV** | **R1** | **R2** | **R3** | **R4** | **R5** | **R6** | **R7** |
| **Strong** | 7 | 8 | 8 | 7 | 9 | 7 | 9 | 7 | 7 | 12 | 9 | 9 | 9 | 11 | 6 | 11 | 9 | 10 | 8 | 8 |
| **Delicate** | 4 | 7 | 5 | 4 | 7 | 5 | 4 | 5 | 3 | 4 | 6 | 3 | 6 | 5 | 6 | 5 | 3 | 4 | 5 | 5 |
| **Balanced** | 5 | 5 | 8 | 6 | 7 | 6 | 6 | 6 | 5 | 4 | 11 | 5 | 8 | 7 | 7 | 7 | 7 | 8 | 8 | 8 |
| **Long aftertaste** | 7 | 6 | 6 | 7 | 8 | 8 | 9 | 8 | 9 | 12 | 6 | 10 | 9 | 9 | 8 | 7 | 7 | 9 | 7 | 9 |
| **Tobacco** | 4 | 6 | 6 | 6 | 6 | 4 | 5 | 3 | 5 | 3 | 3 | 6 | 6 | 6 | 4 | 4 | 5 | 4 | 5 | 6 |
| **Caramel** | 1 | 1 | 2 | 2 | 1 | 0 | 1 | 1 | 1 | 1 | 0 | 0 | 0 | 0 | 1 | 0 | 0 | 0 | 0 | 0 |
| **Licorice** | 5 | 6 | 6 | 3 | 3 | 5 | 4 | 5 | 5 | 4 | 4 | 3 | 1 | 3 | 3 | 3 | 2 | 1 | 3 | 5 |
| **Chocolate** | 2 | 3 | 1 | 3 | 3 | 0 | 2 | 0 | 2 | 0 | 2 | 2 | 2 | 1 | 1 | 1 | 1 | 2 | 2 | 2 |
| **Nutty** | 2 | 1 | 3 | 5 | 2 | 2 | 3 | 1 | 2 | 1 | 4 | 1 | 2 | 4 | 2 | 3 | 3 | 3 | 1 | 4 |
| **Roasted** | 7 | 10 | 10 | 8 | 6 | 8 | 6 | 11 | 9 | 8 | 9 | 7 | 11 | 11 | 10 | 13 | 11 | 12 | 12 | 11 |
| **Burnt** | 10 | 8 | 8 | 11 | 7 | 9 | 11 | 10 | 10 | 12 | 7 | 10 | 9 | 11 | 13 | 13 | 13 | 12 | 8 | 8 |
| **Earthy** | 6 | 9 | 7 | 6 | 8 | 8 | 8 | 7 | 7 | 6 | 6 | 6 | 4 | 3 | 6 | 6 | 8 | 8 | 8 | 4 |
| **Grassy ***** | 4 | 4 | 4 | 3 | 5 | 8 | 5 | 6 | 4 | 3 | 6 | 6 | 5 | 1 | 2 | 0 | 2 | 3 | 3 | 3 |
| **Citrus **** | 2 | 0 | 0 | 0 | 1 | 1 | 0 | 2 | 0 | 0 | 1 | 0 | 0 | 0 | 0 | 0 | 0 | 0 | 1 | 1 |
| **Bitter** | 17 | 12 | 13 | 15 | 14 | 14 | 14 | 14 | 16 | 16 | 13 | 16 | 14 | 16 | 15 | 14 | 16 | 13 | 15 | 14 |
| **Sweet** | 2 | 1 | 2 | 3 | 2 | 1 | 3 | 1 | 2 | 2 | 3 | 2 | 3 | 3 | 2 | 1 | 1 | 0 | 2 | 2 |
| **Acidic *** | 6 | 6 | 8 | 6 | 6 | 6 | 4 | 5 | 7 | 5 | 2 | 5 | 3 | 4 | 3 | 5 | 3 | 4 | 5 | 3 |
| **Astringent** | 7 | 7 | 5 | 6 | 5 | 5 | 6 | 6 | 7 | 6 | 6 | 8 | 7 | 6 | 9 | 6 | 7 | 7 | 7 | 5 |

**Table S8.** Sensory attributes with significantly difference according to citation frequencies (%).

* indicates significant differences between coffee samples according to Cochran’s Q test at p≤0.05
** indicates significant differences between coffee samples according to Cochran’s Q test at p≤0.01
*** indicates significant differences between coffee samples according to Cochran’s Q test at p≤0.001
